# Starfish-inspired wearable bioelectronic systems for physiological signal monitoring during motion and real-time heart disease diagnosis

**DOI:** 10.1126/sciadv.adv2406

**Published:** 2025-04-02

**Authors:** Sicheng Chen, Qunle Ouyang, Xianglin Meng, Yibo Yang, Can Li, Xuanbo Miao, Zehua Chen, Ganggang Zhao, Yaguo Lei, Bernard Ghanem, Sandeep Gautam, Jianlin Cheng, Zheng Yan

**Affiliations:** ^1^ Department of Chemical and Biomedical Engineering, University of Missouri, Columbia, MO, USA.; ^2^ Department of Mechanical and Aerospace Engineering, University of Missouri, Columbia, MO, USA.; ^3^ Department of Critical Care Medicine, The First Affiliated Hospital of Harbin Medical University, Harbin, China.; ^4^ King Abdullah University of Science and Technology, Thuwal, Kingdom of Saudi Arabia.; ^5^ DoorDash Inc., San Francisco, CA, USA.; ^6^ Mechanical Engineering College, Xi’an Jiaotong University, Xi’an, Shaanxi, China.; ^7^ Division of Cardiovascular Medicine, University of Missouri, Columbia, MO, USA.; ^8^ Department of Electrical Engineering and Computer Science, University of Missouri, Columbia, MO, USA.; ^9^ NextGen Precision Health, University of Missouri, Columbia, MO, USA.

## Abstract

Soft bioelectronics enable noninvasive, continuous monitoring of physiological signals, essential for precision health care. However, capturing biosignals during physical activity, particularly biomechanical signals like cardiac mechanics, remains challenging due to motion-induced interference. Inspired by starfish’s pentaradial symmetry, we introduce a starfish-like wearable bioelectronic system designed for high-fidelity signal monitoring during movement. The device, featuring five flexible, free-standing sensing arms connected to a central electronic hub, substantially reduces mechanical interference and enables high-fidelity acquisition of cardiac electrical (electrocardiogram) and mechanical (seismocardiogram and gyrocardiogram) signals during motion when coupled with signal compensation and machine learning. Using these three cardiac signal types as inputs, machine learning models deployed on smart devices achieve real-time, high-accuracy (more than 91%) diagnoses of heart conditions such as atrial fibrillation, myocardial infarction, and heart failure. These findings open previously undiscovered avenues by leveraging bioinspired device concepts combined with cutting-edge data science to boost bioelectronic performance and diagnostic precision.

## Teaser

Starfish-inspired wearable bioelectronics enable high-fidelity cardiac monitoring and AI-driven diagnosis of heart diseases.

## INTRODUCTION

In nature, starfish are characterized by a unique fivefold radial symmetry, which is not only a defining feature but also fundamental to their biology (*1*, *2*). This pentaradial structure allows starfish to move and turn in any direction through the independent operation of their arms. Each arm can function independently and sense its surroundings without interfering with the others, showcasing the advantage of this structure. Emerging wearable soft bioelectronics offer a minimally invasive means of continuously monitoring a spectrum of physiological signals from the human body, with wide-ranging potential applications in human precision health care, soft robotics, augmented reality/virtual reality, human-machine interfaces, and many others (*3*–*8*). While state-of-the-art soft bioelectronics boast high flexibility and advanced integration of various components (*9*–*11*), their typical design—incorporating sensing elements, data acquisition modules, and conductive traces onto a single substrate—poses limitations. This monolithic configuration constrains their ability to effectively mitigate mechanical interference, thereby compromising the high-fidelity capture of biosignals during dynamic bodily movements and restricting their practical applications in real-life scenarios. Drawing inspiration from starfish, we propose that a five-arm, pentaradial device configuration is optimal for minimizing mechanical interference, which can serve as the foundation for enabling high-fidelity recording of physiological signals.

A variety of biosignals can be detected from the human body using wearable soft bioelectronics, offering valuable insights into health status, with cardiac signals being among the most critical (*12*–*14*). Globally, heart disease is responsible for 17.9 million deaths each year, a figure expected to rise to 22.2 million by 2030 (*15*). Many heart conditions, which can emerge during everyday activities, are treatable with early detection and timely intervention (*16*), thereby making ambulatory cardiac monitoring essential for improving patient outcomes and reducing mortality. Now, monitoring cardiac electrical functions by electrocardiogram (ECG) is widely used in clinical practice and is pivotal in diagnosing various heart diseases (*17*–*19*). Simultaneous measurements of cardiac mechanical activities, such as seismocardiogram (SCG) and gyrocardiogram (GCG) that track the translational and rotational components of cardiac-induced chest vibrations, can offer critical information that ECG alone cannot capture (*20*–*22*). These measurements are particularly useful for detecting complications like atrial fibrillation (A-Fib), coronary heart disease, heart failure (HF), myocardial infarction (MI), and hemorrhage (*23*–*25*). Recent advances in soft bioelectronics have enabled wearable heart monitors capable of continuously monitoring ECG, SCG, GCG, or combinations thereof (*26*–*31*). However, the efficacy of the existing devices is largely confined to periods of rest, as physical activities can introduce noise in the form of motion artifacts, particularly in cardiac mechanical signals, limiting their utility for continuous monitoring of cardiac function during everyday activities.

This work introduces a starfish-inspired soft bioelectronic system designed to capture high-fidelity cardiac electrical and mechanical signals during motion. The collected data are wirelessly transmitted via Bluetooth and in situ processed with machine learning (ML) algorithms deployed on smartphones for real-time heart health evaluation. This work represents notable progress in the fields of soft bioelectronics and telemedicine. First, our research harnesses the morphological brilliance of starfish to reshape the device configuration of soft bioelectronics, minimizing mechanical interference. This bioinspired approach establishes a solid foundation for next-generation soft bioelectronic systems capable of capturing high-fidelity biosignals, even under dynamic bodily conditions. Second, the starfish-like wearable device integrates signal compensation with ML-enabled motion recognition and adaptive filtering to achieve high-fidelity recording of cardiac electrical (ECG) and mechanical (SCG and GCG) signals during physical activities. Furthermore, our human studies demonstrate that incorporating all three signal types as input features in ML models achieves an accuracy rate exceeding 91% for real-time heart disease diagnosis, including A-Fib, MI, and HF. This significantly surpasses models using one or two signal types. In addition, the starfish-like device is lightweight (~1.7 g), highly integrated, and features wireless data transmission, remote charging and powering, edge computing, and waterproofing, enabling continuous, real-time heart health monitoring throughout daily activities. Collectively, these advancements in bioinspired device design, multimodal signal monitoring, advanced system integration, and real-time, high-accuracy heart disease diagnosis mark a significant leap in soft bioelectronics and telemedicine. A comprehensive comparison of our device with state-of-the-art wearable heart monitors is provided in table S1. The insights and design principles presented in this work can be extended to the development of other wearable bioelectronic systems with customized functionalities tailored to specific health conditions, further advancing precision health care.

## RESULTS

### Starfish-inspired design and analysis

**Fig. 1.**
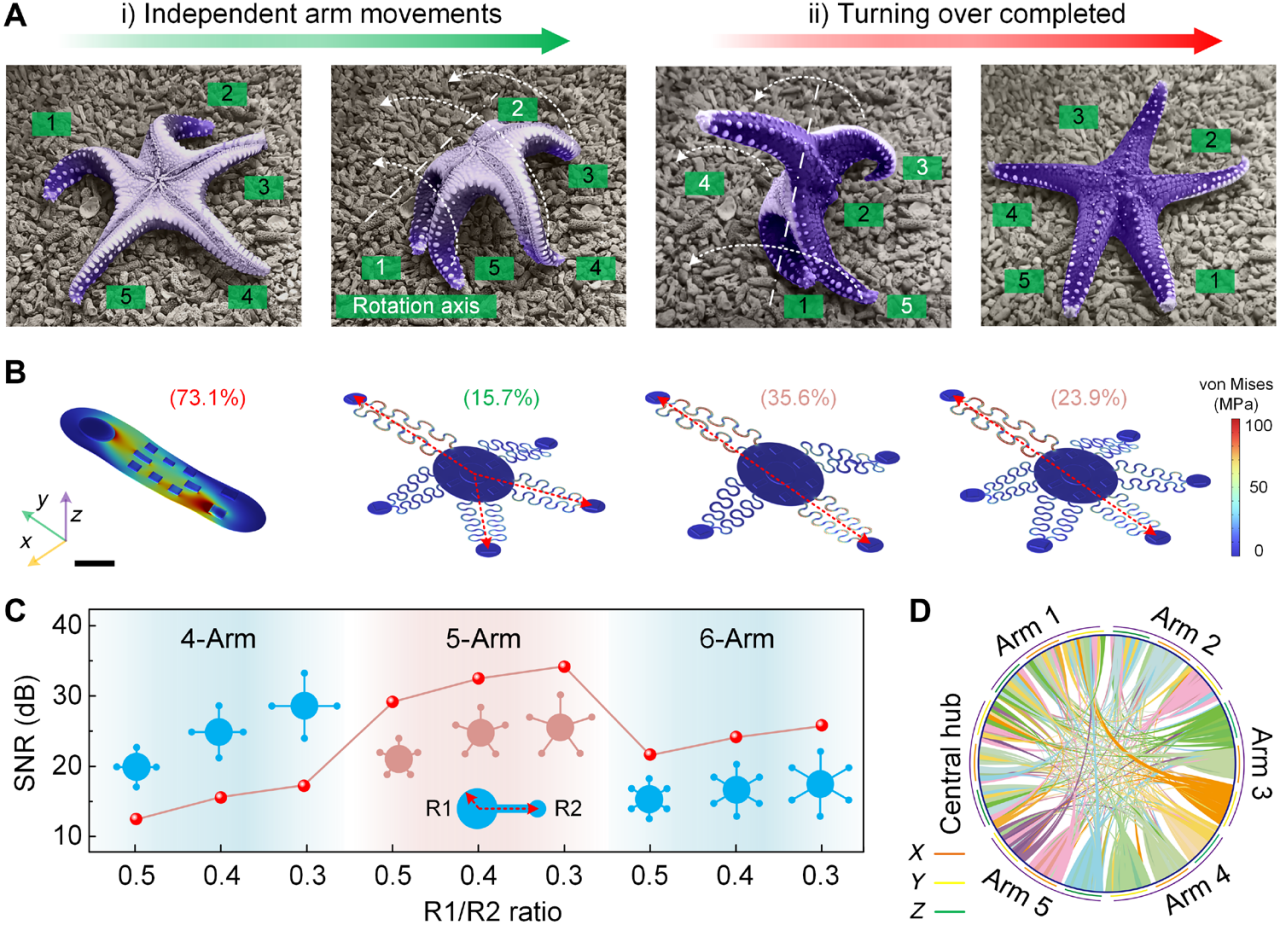
Starfish-inspired design and analysis. (**A**) Optical images illustrating the process of a starfish flipping over via independent arm movements. The starfish anchors arms 1 and 2 to serve as a pivot for rotation, while arms 3 and 5 lift the body in preparation for landing on arm 4. This coordinated yet independent movement enables the starfish to adapt effectively to its environment. (**B**) FEA simulations of stress distribution under a 10-mm displacement applied to top-left corners of various soft bioelectronic device configurations, including a traditional monolithic design and starfish-inspired designs with four, five, and six arms. The most pronounced stress coupling occurs along the diagonal axes in all configurations, with the five-arm configuration exhibiting the lowest stress coupling coefficient at 15.7%, significantly lower than four-arm (35.6%), six-arm (23.9%), and monolithic (73.1%) designs. Color bar, stress distribution. Scale bar, 10 mm. (**C**) Relationship between the SNR and the R1/R2 ratio, where R1 represents the radius of the central hub and R2 is the extended length of the sensing pad. The five-arm configuration with an R1/R2 ratio of 0.3 demonstrates substantial SNR improvements over other configurations. The inset shows the definitions of R1 and R2. Detailed SNR calculations are provided in text S1. (**D**) Network diagram illustrating the coupling relationships among the five arms and the central hub of the starfish-like wearable device during motion. The diagram highlights the reduced coupling constraints in the 3D displacement of each arm, which allows for more flexible motion in all directions, enhancing the device’s capability to collect high-fidelity biosignals during activity. The *x*, *y*, and *z* represent 3D spatial coordinates. Related FEA simulations for the four-arm, five-arm, six-arm, and monolithic designs are provided in fig. S3.

Figure 1A illustrates the coordinated yet independent movements of a starfish’s five arms during the process of flipping over. Initially, arms 1 and 2 would contract to serve as the axis of rotation, while arms 3 and 5 support the body as it lifts arm 4 in preparation for landing. This coordinated action enables the starfish to move in any direction, showcasing its adaptability. Inspired by starfish, we propose a five-arm, pentaradial device configuration and compare it with traditional monolithic designs, in which all components are integrated onto a single patch, as well as to four-arm and six-arm configurations. Finite element analysis (FEA) simulations (Fig. 1B) reveal the stress distribution under a 10-mm displacement applied to top-left corners of various soft bioelectronic device configurations. The highest stress coupling occurs along the diagonal axes in all configurations. The ratio of the average stress on the diagonal axes to the average stress on the arm actively undergoing displacement is defined as the stress coupling coefficient (text S1). While serpentine-based designs have been used to address mechanical mismatches between skin and devices, the specific device configuration is a crucial factor to mitigate mechanical coupling. FEA results (Fig. 1B and fig. S1A) show that the five-arm design achieves the lowest stress coupling coefficient at 15.7%, significantly lower than the four-arm (35.6%), six-arm (23.9%), and monolithic (73.1%) designs. This underscores the effectiveness of the starfish-inspired configuration in minimizing mechanical interference. Furthermore, incorporating a monolithic substrate into these five-, four-, and six-arm designs significantly increases the device’s coupling coefficient (fig. S1B), demonstrating the superiority of the starfish-inspired configuration over traditional monolithic designs. This five-arm structure can effectively bifurcate the coupling path, reducing mechanical coupling. Mechanical vibration experiments conducted on artificial skins (fig. S2 and movie S1) further validate the five-arm design’s ability to reduce mechanical coupling. To further assess performance, we convert the coupling coefficient into signal-to-noise ratio (SNR) and evaluate the influence of different arm configurations (four, five, and six arms) and arm size parameters (the ratio of the central hub’s radius to the extended length of the sensing pad, R1/R2) on SNR. FEA results indicate that arm configuration has a greater impact on SNR than arm size (Fig. 1C and fig. S3). For example, reducing the R1/R2 ratio from 0.5 to 0.3 in the four-arm configuration increases SNR by 5 dB (from 12 to 17 dB), while the five-arm configuration achieves an SNR enhancement of 17 dB (from 12 to 29 dB). On the basis of these results, the five-arm configuration with an R1/R2 ratio of 0.3 is selected for the starfish-like device design. Figure 1D presents a network diagram illustrating the coupling relationships among the arms and central hub of the device during movement. Unlike the monolithic design, which shows overall coupling within a plane during motion (fig. S4), the three-dimensional (3D) displacement of each arm in the five-arm structure experiences minimal coupling constraints from other arms, allowing for greater flexibility in all directions. This flexibility enhances the ability of the starfish-like wearable device to capture high-fidelity signals during human motion.

### System design and algorithms of the starfish-like wearable device for trimodal cardiac monitoring

**Fig. 2.**
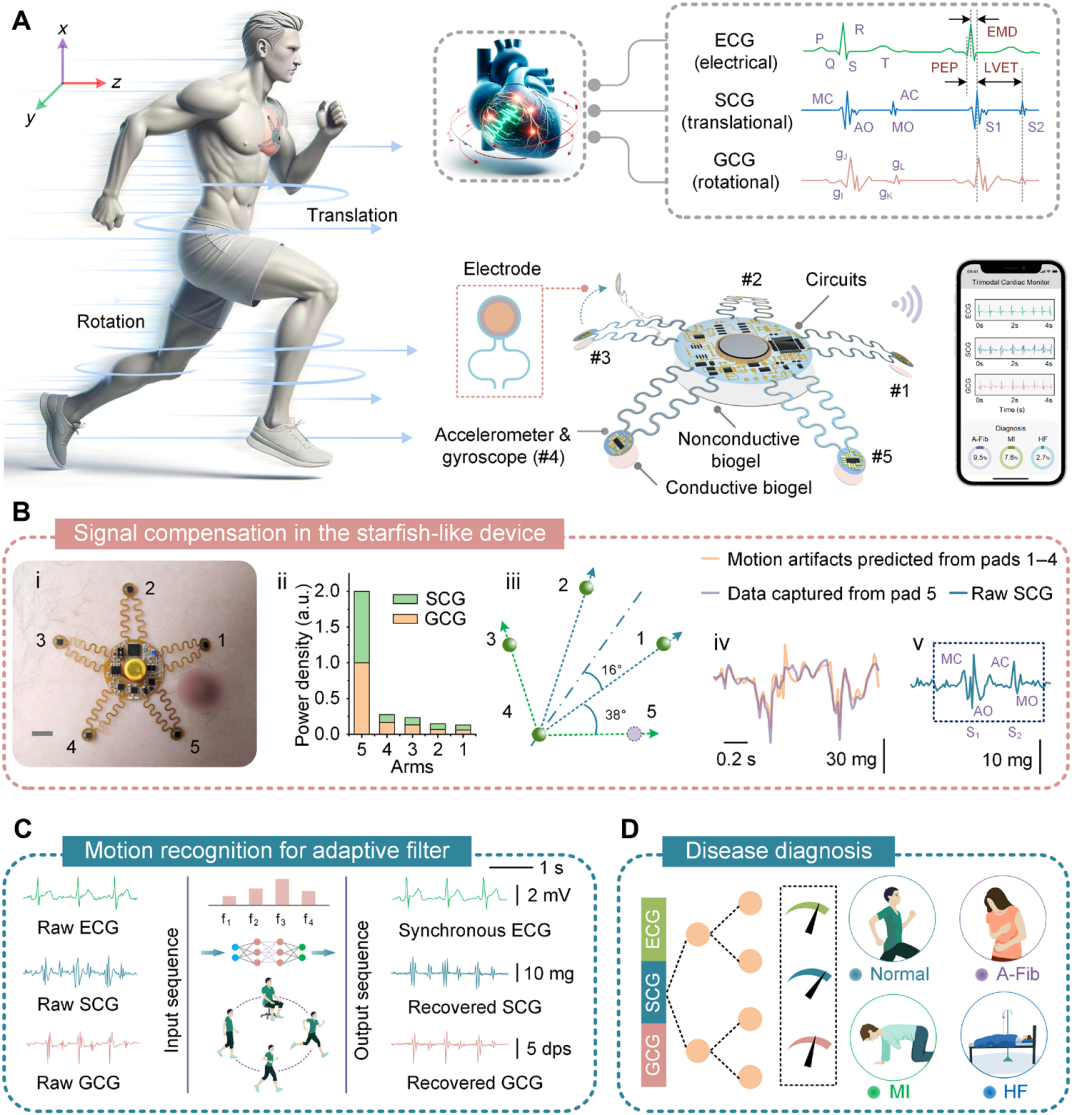
System design and algorithms of the starfish-like wearable device for trimodal cardiac monitoring. (**A**) Schematic illustration of the starfish-like device for trimodal cardiac monitoring during motion. P, atrial depolarization; QRS, ventricular depolarization; T, ventricular repolarization; MC, mitral valve closure; AO, aortic valve opening; AC, aortic valve closure; MO, mitral valve opening; S1, first heart sound; S2, second heart sound; g_I_, isovolumetric contraction; g_J_, aortic valve opening; g_K_, aortic valve closure; g_L_, mitral valve opening; EMD, electromechanical delay; PEP, pre-ejection period; LVET, left ventricular ejection time. (**B**) Signal compensation for cardiac mechanics monitoring using the starfish-like device. [(B), i] Optical image showing the device applied to the chest skin, with the five strategically positioned sensing pads; [(B), ii] power density comparison of SCG and GCG signals recorded from the five sensing pads; [(B), iii] motion artifacts on sensing pad 5 predicted from pads 1 to 4 through vector synthesis; [(B), iv] motion artifacts (orange) on sensing pad 5, which are derived from signals recorded by pads 1 to 4, alongside signals (purple) recorded from pad 5 that contain both motion artifacts and cardiac mechanical signals; [(B), v] the resulting raw SCG signal on sensing pad 5 after subtracting motion artifacts. Scale bar, 1 cm. (**C**) Conceptual illustration of ML-enabled motion recognition and adaptive filtering. The model classifies motion states and applies corresponding filters to reduce motion artifacts in real time. Further details are provided in Figs. 3 and 4. (**D**) Conceptual framework for ML-enabled heart disease diagnosis using ECG, SCG, and GCG signals as input features. Labeled data from healthy individuals and patients with cardiac diseases are input into a convolutional neural network, which extracts features and distinguishes between normal and abnormal heart conditions. Additional details are provided in Fig. 5. a.u., arbitrary units.

Inspired by the unique features of starfish, we develop an exemplary wearable device for trimodal cardiac monitoring and heart disease diagnosis (Fig. 2A). This device enables high-fidelity recording of cardiac electrical (ECG) and mechanical (SCG and GCG) signals during physical activity. By analyzing the characteristic peak groups and their relative positions within the recorded electrical and mechanical waveforms (text S2), the device provides comprehensive assessments of heart health. The device is built on a 25-μm-thick polyimide (PI) substrate, with copper traces serving as conductive pathways. The pentaradial design features five serpentine arms, each equipped with independent sensing elements at the tips (i.e., sensing pads), all connected to a central electronic hub for data processing and wireless transmission. To capture SCG and GCG signals while monitoring physical activity, the device integrates five high-performance commercial accelerometer-gyroscope sensing units (BMI270) on the sensing pads. These sensors, along with other off-the-shelf electronic components (table S2), are mounted on the top surface of the device, avoiding direct skin contact. Each sensing pad is equipped with a gold-plated copper electrode on the backside, connected to the circuitry through vertical vias in PI substrates, enabling ECG recording upon skin contact. The skin interface layer is composed of conductive biogels for the five sensing pads and nonconductive biogels for the central hub, while the serpentine arms remain free-standing. Weighing approximately 1.7 g (with ~0.7 g for the device and ~1 g for the battery), the wearable system is virtually imperceptible when worn. The collected signals are processed by a 32-bit microcontroller and wirelessly transmitted (fig. S5 and text S3), with analyzed data visually displayed on mobile devices. Trained ML algorithms, deployed on mobile devices, perform edge computing to analyze recorded data, enabling real-time diagnosis of heart conditions. The device operates with a sampling rate of 200 Hz, capturing all channels (mechanical and electrical signals) simultaneously. To optimize power consumption, a data buffering strategy is implemented to store and preprocess the acquired data before transmission. This approach reduces the transmission frequency to 10 transmissions per second, significantly decreasing the power required for wireless data transfer while ensuring high-fidelity signal acquisition and real-time applicability. The system is powered by an onboard rechargeable battery (LIR1240, 50 mAh), supplemented by wireless charging coils. This setup allows for continuous operation of approximately 8 hours, with an average power consumption of ~6 mA. The remote power management system not only recharges the device battery in various conditions, such as using a commercial charger, during on-body use, and even underwater, but can also supply power directly to the device without a battery, ensuring its continued operation (fig. S6 and movie S2).

Thanks to the conductive, adhesive biogel used as the electrode-skin interface (fig. S7) and the mechanically decoupled starfish-like device design (Fig. 1B), the device achieves high-fidelity ECG recordings across various motion states, maintaining an SNR of approximately 35 dB even during running (fig. S8). To ensure high-quality recording of cardiac mechanical signals, we further use signal compensation (Fig. 2B) and ML-enabled motion recognition with adaptive filtering (Fig. 2C). The optical image in Fig. 2Bi illustrates the strategic placement of the device, with sensing pad 5 positioned near the heart to capture robust cardiac mechanical signals. The remaining four sensing pads, located farther from the heart, primarily monitor human motion using embedded accelerometers and gyroscopes. As demonstrated in Fig. 2Bii, the SCG and GCG power densities recorded from sensing pad 5 (the acquisition channel) are significantly higher (~5 to 15 times) than those recorded from the accelerometers and gyroscopes in sensing pads 1 to 4 (compensation channels) during rest. During motion, sensing pad 5 captures strong cardiac mechanical signals along with motion artifacts, while sensing pads 1 to 4 primarily record motion artifacts. The motion artifact components can be predicted from the signals of pads 1 to 4 using vector synthesis methods (Fig. 2Biii). Detailed processing steps are further explained in text S4 and fig. S9. This enables us to isolate and subtract motion-related noise from the signals recorded by sensing pad 5 using the data from sensing pads 1 to 4, thus extracting raw cardiac mechanical signals. Our results in Fig. 2B (iv) and (v) and fig. S10 indicate the efficacy of the signal compensation strategy, revealing distinct feature peaks in raw SCG and GCG signals recorded during running. Despite these efforts, some residual motion artifacts persist. To further address this, we develop a neural convolutional network model capable of real-time motion state analysis and adaptive filtering to reduce artifacts and improve signal quality (Fig. 2C). This model classifies motion states into categories such as stationary, walking, jogging, and running, applying corresponding filters to adaptively process the raw cardiac mechanical signals. We further analyze the filtered data to explore the correlation between cardiac electrical and mechanical signals and various heart conditions (Fig. 2D). Labeled signals from healthy individuals and patients with diagnosed cardiac conditions are used as inputs for diagnostic models. These signals are sequentially processed through a layered convolutional neural network, with input sequences extracted as feature sets. The trained classifier can effectively distinguish between normal and abnormal samples, providing early warnings to users with abnormal cardiac signals. More details are provided in the following sections.

### ML-driven motion recognition

**Fig. 3.**
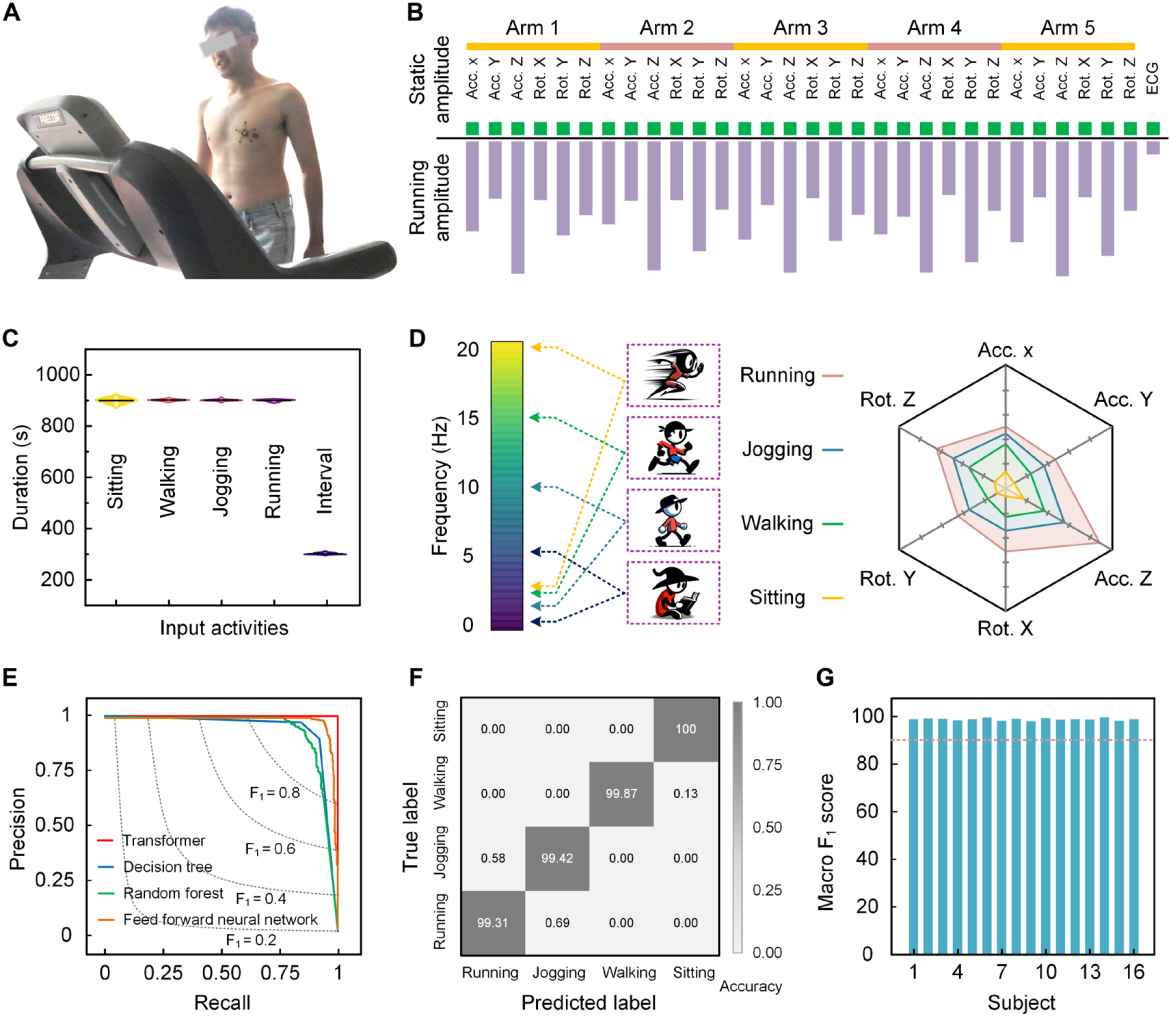
ML-driven motion state recognition. (**A**) The starfish-like device worn on the chest of a human subject during running. (**B**) Relative power density of signals collected during static (sitting) and running. All 30 channels show a significant increase in signal energy during running across all dimensions, normalized to the energy in the sitting state. (**C**) Motion status data collected as input for the ML model, with contributions from 16 human subjects, resulting in more than 576,000 s of valid input data. (**D**) Initial analysis of mechanical signals collected across various motion states, showing that as activity intensity escalates from sitting to running, the higher frequency components of mechanical signals increase accordingly. Both translational and rotational energy levels rise significantly. (**E**) Precision-recall curves, comparing the performance of different ML models for motion recognition. (**F**) Confusion matrix, illustrating classification accuracy for predicting each motion state in the test set. (**G**) Overall classification accuracy for motion state based on the macro-averaged F1 score across human subjects, showing accuracy rates above 95%.

Empowered by the capability of collecting signals during motion, the starfish-like wearable device enables long-term, real-time, continuous monitoring of daily physical activities. As shown in Fig. 3A, the device can adhere securely to the volunteer’s chest, even during running. Figure 3B illustrates the collected acceleration (Acc. X, Acc. Y, and Acc. Z) and rotation (Rot. X, Rot. Y, and Rot. Z) signals across the *x*, *y*, and *z* axes from each arm, encompassing 30 channels in total. Normalized to the energy level of each channel during the static state (i.e., sitting), the energy of recorded mechanical signals in each dimension increases significantly during running, with the *Z*-direction acceleration increasing more than 10-fold. The overall signals display rhythmic, stride-synchronized periodic patterns (fig. S11). To evaluate these motion-related features and prepare for the removal of motion artifacts from biomechanical signals, we further develop an ML model to extract features and deconvolve the connections between biomechanical signals and motion signals collected by our device. The model is trained on four predefined motion states: sitting, walking, jogging (2 mph), and running (5 mph), using data from 16 human subjects, contributing more than 576,000 s of valid input data (Fig. 3C and fig. S12). Short rest intervals are excluded from the model as inputs. The ML model uses a 10-s sliding time window for signal segmentation, with a sampling frequency of 200 Hz, resulting in 2000 sample points per segment. These segments are processed with a transformer model to determine hidden states and extract input sequences as feature sets (fig. S13). The model uses temporal information within each time window to improve motion predictions based on contextual data.

Before feeding raw signals into the ML model, we conducted an initial analysis of mechanical signals under various motions. With short-time Fourier transforms, we identify the frequency and relative intensity as key characteristics of mechanical signals across different motion states (fig. S14). As activity intensity increases from sitting to running, the components of mechanical signals at the higher frequencies (~20 Hz) correspondingly increase (Fig. 3D). In addition, both translational and rotational energy levels significantly increase, with the most notable rise observed in the *Z*-direction acceleration. In addition to frequency and relative intensity, identifying characteristics such as coordination between rotational and translational signals and gait rhythm is difficult to observe directly, so we use an ML model to classify raw mechanical signals captured using this device. Several ML models are evaluated, and the transformer model outperforms others, including decision tree, random forest, and neural network composed of fully connected layers (Fig. 3E and fig. S15). Combined with features extracted from both translational and rotational motion data, the transformer model achieves accuracies of 100, 99.87, 99.42, and 99.31% for sitting, walking, jogging, and running, respectively (Fig. 3F and fig. S16). Moreover, the transformer model consistently delivers accuracies exceeding 95% across different human subjects (Fig. 3G). To assess the contribution of mechanical signals and derived features from each dimension to motion state recognition, we conduct a Shapley additive explanation (SHAP) analysis to evaluate feature importance (fig. S17). The SHAP analysis assigns a Shapley value to each feature, quantifying its contribution to the model’s predictions. This approach enables the identification of key features, such as acceleration or rotational components from specific axes, that have the most significant impact on motion state classification. These results confirm that our ML model reliably establishes correlations between biomechanical signals and motion states, leveraging frequency domain and intensity information to achieve accurate classifications.

### Motion-adaptive data processing of heart biomechanics

**Fig. 4.**
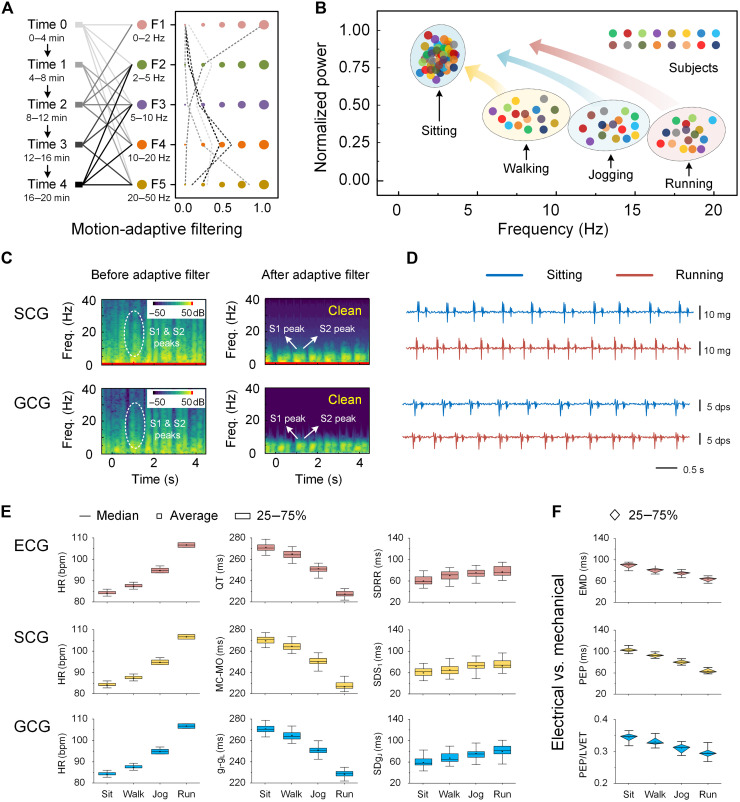
Motion-adaptive data processing of heart biomechanics. (**A**) Adaptive combination filtering applied to compensated cardiac biomechanical signals within each 10-s interval. During each interval, the human motion state is classified into composite probabilities for sitting, walking, jogging, and running, with each state corresponding to a specific combination filter. (**B**) Uniform transformation of cardiac biomechanical signals across different frequency ranges during motion, resulting in high-quality signals comparable to those obtained under stationary conditions. (**C**) Time-frequency plots of heart biosignals before and after applying adaptive filters. These filters can effectively preserve peak positions while narrowing the primary energy frequency range, enhancing the clarity and quality of the signal display. (**D**) Heart biomechanical signals (SCG and GCG) recorded from a human subject in both running and sitting states. (**E**) HR, QT, SDRR, and corresponding parameters (mitral valve closure–mitral valve opening, SDS_1_, g_I_-g_L_, and SDg_J_) monitored under various motion states using the starfish-like wearable device. The data indicate an increase in HR and a decrease in both QT and SDRR as exercise intensity increases. HR, heart rate; QT, QT interval; SDRR, SD of RR intervals; bpm, beats per minute. (**F**) EMD, PEP, and PEP/LVET parameters measured across different motion conditions, reflecting the relative relationship between heart mechanical and electrical biosignals.

Accurate recognition of different motion states is essential to the motion artifact elimination algorithm, as motion artifacts exhibit distinct characteristics depending on the specific motion state. Precise identification of the body’s current state of motion is crucial for applying the appropriate filter. Leveraging real-time motion states predicted by the ML model, we apply tailored combination filters to the corresponding compensated cardiac biomechanical signals within each 10-s interval (Fig. 4A). During each time interval, the motion state is classified into composite probabilities for sitting, walking, jogging, and running, with each state corresponding to a specific filter. By recombining these filters according to the composite probabilities, an adaptive combination filter is applied in real time. By first compensating for heart biomechanical signals collected from our device (Fig. 2B), then identifying motion states with the ML-driven motion recognition model (Fig. 3), and lastly applying adaptive combination filters based on the recognized motion states (fig. S18), we can collect high-quality cardiac biomechanical signals during movement, comparable to those obtained in a stationary state (Fig. 4B). Figure 4C presents the time-frequency plots of cardiac biomechanical signals before and after applying the adaptive filters. These filters not only preserve peak positions but also narrow the primary energy frequency range, significantly improving the clarity and quality of the signals. These high-quality signals can be displayed in real-time on smart devices, allowing users to promptly access cardiovascular health information during various motion states and everyday activities. Figure 4D shows SCG and GCG signals from a subject in both running and sitting states. The concurrently recorded ECG signals are provided in fig. S19. Even during various motion sates, the recorded cardiac biosignals maintain high SNRs comparable to that of a stationary state while clearly preserving distinct feature peaks (fig. S20). This ability to decouple motion artifacts is not only a result of signal compensation and motion-adaptive filtering but also due to the bioinspired starfish-like device configuration. In contrast, traditional monolithic wearable devices suffer from significant signal distortion and a rapid decline in SNR during physical activity (fig. S21 and movie S3).

The feature peaks in ECG signals provide essential cardiac health information, including heart rate (HR), QT interval, and SD of RR intervals (SDRR). These metrics reflect heart rhythm, HR variability, and electrical activity duration (*32*). In SCG signals, mitral valve closure–mitral valve opening and the SD of the S1 peak correspond to QT and SDRR, respectively, while in GCG signals, g_I_-g_L_ and SDg_J_ are used for the same metrics. This starfish-like device successfully monitors these parameters across various motion states (Fig. 4E). As exercise intensity increases, HR rises while QT and SDRR decrease. This is due to the body’s increased oxygen demand during physical activity, which activates the sympathetic nervous system, elevating HR to supply more oxygenated blood to the muscles. As HR increases, the cardiac cycle shortens, reducing the QT interval to accommodate faster beats. In addition, SDRR decreases as the HR becomes more regular due to heightened sympathetic activity. Compared to the information from ECG, SCG, or GCG signals alone, the combined data from cardiac mechanical and electrical signals provides a deeper insight into heart health. Parameters such as electromechanical delay (EMD) and pre-ejection period (PEP) capture the temporal offsets between mechanical and electrical signal peaks, as well as the speed at which the heart initiates mechanical actions following electrical activity (text S5). Left ventricular ejection time (LVET) measures the duration of blood ejection from the left ventricle into the aorta, while the PEP/LVET ratio serves as a valuable index for assessing cardiac contractility and overall heart function (*33*). Typically, a lower PEP/LVET ratio indicates more efficient cardiac performance. Variations in these parameters can reveal underlying cardiac pathologies or altered physiological states, providing valuable insights for diagnosing and monitoring heart conditions (*34*). Measurements of EMD, PEP, and the PEP/LVET ratio across different motion states in healthy subjects, as recorded by the starfish-like wearable device, are shown in Fig. 4F. As exercise intensity increases, these parameters decrease. EMD shortens because of sympathetic nervous system activation, which accelerates electrical impulse conduction and enhances myocardial contractility during exercise. PEP decreases as increased contractility, driven by sympathetic stimulation, enables the heart to build pressure and eject blood more rapidly to meet heightened metabolic demands. LVET also shortens as the HR increases, reducing the duration of the overall cardiac cycle. Since PEP decreases more rapidly than LVET during exercise, the PEP/LVET ratio declines, indicating that the heart spends relatively less time in the pre-ejection phase compared to the ejection phase—reflecting improved efficiency in contracting and ejecting blood during physical activity.

### Real-time, high-accuracy heart disease diagnosis

**Fig. 5.**
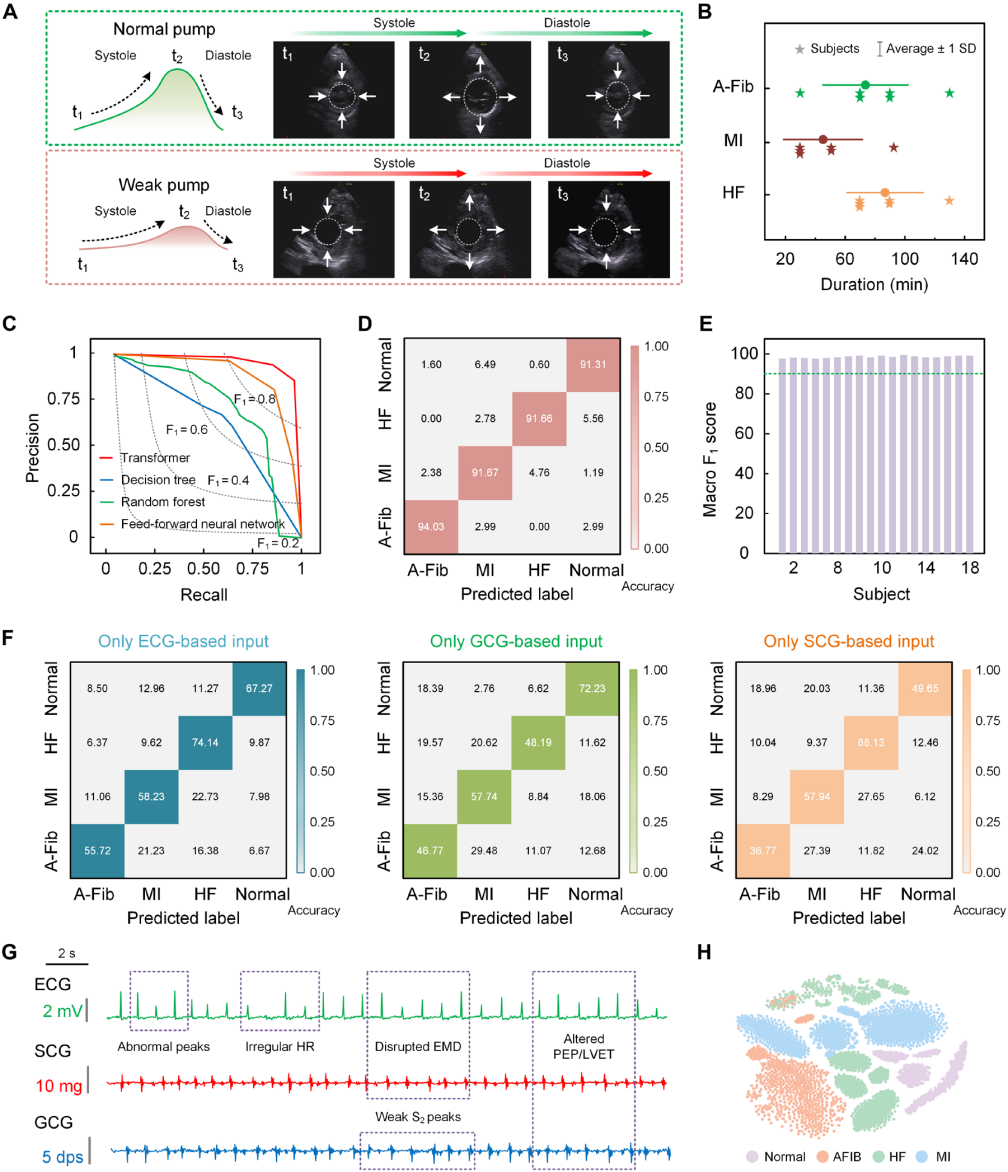
Real-time, high-accuracy heart disease diagnosis. (**A**) Ultrasound imaging illustrating the differences in heart pumping between a patient with early-stage HF and a healthy individual. In the HF patient, both the contraction and relaxation amplitudes are below normal levels. (**B**) Data collected using the starfish-like wearable device for three heart conditions: A-Fib, MI, and HF, with a total collection time exceeding 20 hours. (**C**) Precision-recall curves, comparing the performance of different ML models for heart disease diagnosis. (**D**) Confusion matrix, demonstrating the classification accuracy for predicting each type of heart disease and normal signals in the test set. During use, the starfish-like device can display the real-time classification probabilities on smart devices. (**E**) Overall classification accuracy for heart health status, based on the macro-averaged F1 score across human subjects, with accuracy exceeding 91%. (**F**) Confusion matrix, comparing the classification performance of using single ECG, SCG, or GCG signals as input. The results demonstrate that combining ECG, SCG, and GCG signals significantly improves classification accuracy. (**G**) Cardiac electrical and mechanical signals collected by the starfish-like wearable device from a HF patient during walking; dps, degrees per second. (**H**) t-Distributed stochastic neighbor embedding plot of the dataset recorded by our device, visually illustrating feature separation among various heart conditions in a 2D space.

Cardiac signals obtained from the starfish-like wearable device can be used for routine monitoring of heart health. In this study, we explore the potential of this device for diagnosing cardiac diseases such as A-Fib, MI, and HF, which are closely associated with abnormalities in the heart’s mechanical contraction and relaxation phases (*35*–*37*). Traditionally, heart disease diagnosis relies on ultrasound imaging to assess cardiac function and evaluate the heart’s pumping efficiency (*38*). For example, Fig. 5A and movie S4 highlight the differences in heart function between a healthy individual and a patient with early-stage HF, showing diminished contraction and relaxation amplitudes in the patient, which result in reduced ejection performance (*39*). We used our device to collect cardiac signals (ECG, SCG, and GCG) from six patients with A-Fib, six with MI, and six with HF, totaling over 20 hours of data (Fig. 5B). These cardiac biosignals, along with those from healthy individuals, are used to train the ML model to classify normal and abnormal signals, facilitating disease diagnosis and early detection. We evaluate several ML models, and the transformer model outperforms alternatives such as decision tree, random forest, and neural network composed of fully connected layers (Fig. 5C and fig. S22). Incorporating features extracted from the combined ECG, SCG, and GCG signals, the transformer model achieves high classification accuracy of 91.31% for normal, 94.03% for A-Fib, 91.67% for MI, and 91.66% for HF (Fig. 5D), outperforming other ML models (fig. S23). During use, this starfish-like device displays real-time classification probabilities of the wearer’s cardiac health and potential disease risks on smart devices, as inferred by the ML model (fig. S24). These probabilities represent the likelihood of specific conditions, including A-Fib, MI, and HF, enabling users to monitor their cardiac status in real time. In addition, the model consistently maintains accuracies exceeding 91% across different individuals (Fig. 5E). Notably, combining three mechanical and electrical signals significantly improves heart disease diagnostic accuracy compared to using individual ECG, SCG, or GCG signals (Fig. 5F) or any two-signal combinations (fig. S25). For instance, the accuracy of distinguishing between normal and abnormal cardiac status using individual ECG, GCG, or SCG signals is only 20, 30, and 40%, respectively. The improved accuracy with combined signals stems from the vital interplay between mechanical and electrical signals in assessing cardiac health. By integrating all three signals into the ML model, a more comprehensive view of the heart’s mechanical response to electrical activation is achieved—an essential indicator of cardiac function. Figure 5G presents cardiac mechanical and electrical biosignals captured by the starfish-like wearable device during walking, from a patient clinically diagnosed with HF. The signals reveal diminished Q and T peaks, irregular HR, disrupted EMD, and altered PEP/LVET parameters, indicative of electrophysiological disturbances. In addition, the patient’s cardiac mechanical activity is significantly impaired, with contraction amplitudes nearly 60% lower than those of a healthy individual. The high-fidelity recording and integration of electrical and mechanical cardiac signals play a crucial role in achieving high diagnostic precision. As a result, the cardiac biosignals captured by our device, along with the extracted features, can effectively classify the heart health status (Fig. 5H). To validate feature extraction, we apply t-distributed stochastic neighbor embedding to project the multidimensional feature space into a 2D representation. Datasets from healthy individuals, as well as those with A-Fib, MI, and HF, naturally form distinct clusters, highlighting the discriminative power of the extracted features.

## DISCUSSION

In this study, we introduce a starfish-inspired wearable bioelectronic system that enables high-fidelity monitoring of biosignals during motion, overcoming the inherent limitations of conventional monolithic soft bioelectronics. Our pentaradial design, derived from the natural mechanics of starfish, effectively mitigates motion-induced artifacts by mechanically decoupling signal acquisition across independent arms. This innovation, combined with ML-driven signal processing, allows for accurate acquisition of both electrical (ECG) and mechanical (SCG and GCG) cardiac signals, even during dynamic activities. Human studies demonstrate that integrating these multimodal biosignals significantly enhances diagnostic accuracy, achieving more than 91% accuracy for various heart conditions—surpassing models that rely on fewer inputs. In addition, this starfish-like device is lightweight, waterproof, supports wireless charging and powering, incorporates edge computing, and is optimized for mass production (fig. S26), making it ideal for continuous, real-time health monitoring in everyday settings. This starfish-like device represents a significant advancement in soft bioelectronics for precision health care, demonstrating the potential of bioinspired device designs and ML-enabled signal processing to enhance signal integrity and improve diagnostic precision in real-time conditions, thus expanding the potential of wearable health technologies.

## MATERIALS AND METHODS

### The starfish-like wearable device fabrication

Starfish-like wearable devices with various arm configurations and monolithic wearable devices are fabricated with the standardized flexible printed circuit board (fPCB) process (*40*, *41*), optimized for mass production. The fPCB, with a total thickness of ~105 μm, is constructed with a 25-μm-thick PI substrate and 12-μm-thick, 150-μm-wide copper conductive traces. To ensure long-term stability, waterproofness, and environmental resistance, the copper traces in areas not designated for landing electronic components are encapsulated with an additional 12.5-μm PI layer, coated with 15-μm-thick epoxy resins. Vertical interconnections (vias) penetrate the PI substrate, connecting copper traces on both sides, minimizing wiring length, reducing signal delay, and optimizing spatial layouts of the circuitry. ECG electrodes are further coated with a 100-nm-thick gold layer over 12-μm-thick copper to enhance long-term stability. Off-the-shelf electronic components listed in table S2, such as the BMI270, processor, wireless charging rectifier, low dropout regulator, and inertial measurement unit, are attached to the designated landing pads using solder pastes (Chip Quik TS391LT). To further enhance waterproofness, all landing pads are coated with a 10-μm-thick layer of ultraviolet-curable adhesive (Norland Optical Adhesive 61).

### Biogel synthesis

The nonconductive biogel is synthesized by dissolving 0.62 g of ε-polylysine [25% (w/v) aqueous solutions, JNC Corp.], 2.5 g of gelatin, and 0.38 g of Na_2_SO_4_ in 10 g of water/glycerol mixture (2:7 by weight). The solution is stirred for 30 min to ensure complete dissolution. Cross-linking is then initiated by adding 1.5 g of borax, followed by stirring for 3 hours at 60°C. The resulting biogel shows solid-like properties below 35°C, transitioning to a liquid-like state above 35°C. For the conductive biogel, water is replaced with 2.29 ml of silver nanowire solutions (AgNW-40, ACS Material). ε-polylysine provides antibacterial properties. In this study, the conductive biogel is applied to the five sensing pads for ECG recording, while the nonconductive biogel is used on the central hub for skin adherence, allowing the five serpentine arms to remain free-standing.

### FEA simulations

To theoretically analyze mechanical responses and optimize device design parameters of various components of the starfish-like wearable device, FEA simulations are conducted using COMSOL Multiphysics 6.1. Four-arm, five-arm, and six-arm configurations, along with monolithic wearable devices and specific structural components, are designed using 3D modeling. The mesh is refined using the COMSOL’s physics-controlled meshing tools, with all objects being meshed into linear hex elements. The minimum mesh size is set to one-fourth the width of the arm to ensure simulation accuracy and convergence. The elastic modulus (*E*) and Poisson’s ratio (ν) are as follows: for copper, *E*_Cu_ = 120 GPa and ν_Cu_ = 0.34; for PI, *E*_PI_ = 3 GPa and ν_Cu_ = 0.38; for gold, *E*_Au_ = 80 GPa and ν_Au_ = 0.42. To ensure consistency in structural design, the edges of each arm in the four-arm, five-arm, and six-arm configurations are created through rotational symmetry. The central hub is set with a diameter of 30 mm, and different arm lengths are parameterized to compare coupling coefficients under various displacement conditions. When arm tips (sensing pads) are constrained or under unique boundary conditions, coupling coefficients are calculated on the basis of relative displacement factors. These coefficients are then converted into SNRs, as detailed in note S1.

### ML models for motion status recognition

All training models are developed using Python (version 3.8), based on data collected from 16 healthy human subjects performing various physical activities, including sitting, walking, jogging, and running. The dataset consists of 57,600 s of recordings from the starfish-like wearable device. The collected signals are segmented using a 10-s sliding window, with each segment containing 2000 sample points, corresponding to the device’s 200-Hz sampling frequency. These segments undergo a preprocessing phase before model evaluations. Four ML models are assessed, including the transformer, decision tree, random forest, and feed-forward neural network, with performance measured by precision-recall curves and F1 scores. The transformer model exhibits superior performance in motion status recognition compared to the other models.

### ML models for heart disease diagnosis

All training models are developed using Python (version 3.8), based on data collected from 18 patients with heart conditions, including A-Fib, MI, and HF. The dataset comprises more than 20 hours of recordings from the starfish-like wearable device. The collected signals are segmented using a 10-s sliding window, with each segment containing 2000 sample points, corresponding to the device’s 200-Hz sampling frequency. These data are mixed with normal data collected from healthy human subjects. Four ML models, including the transformer, decision tree, random forest, and feed-forward neural network, are evaluated using precision-recall curves and F1 scores. The transformer model consistently outperforms other models in heart disease classification. The same model architecture is used for both disease diagnosis and motion recognition tasks (fig. S27). Specifically, a linear layer with an output dimension of 512 transforms the input signals into hidden states, which are then processed through eight Transformer encoder layers. Each Transformer layer includes a multihead self-attention module with four heads and a feedforward module with a dimension of 1024. Following the Transformer layers, a linear classifier maps the extracted features to the output space, where the number of output classes is 5 for motion recognition and 4 for disease diagnosis. Cross-entropy loss is used as the classification loss function. The model is trained using the AdamW optimizer with an initial learning rate of 1 × 10^−4^, betas set to (0.9, 0.999), and a weight decay of 1 × 10^−5^. Training is conducted for 10,000 iterations with a batch size of 1500. Each training sample consists of a time length of 50 timestamps, truncated for training efficiency. For testing, the time length is set to 200 timestamps to ensure comprehensive evaluation.

### Human studies

The starfish-like wearable device evaluation on healthy subjects is conducted in accordance with Institutional Review Board (IRB) #2099478, approved by the University of Missouri-Columbia (MU), USA. Volunteers are recruited from MU students, faculty, and staff. Participants with heart-related conditions are excluded. The device evaluation on patients with heart diseases is conducted under the approval of the First Affiliated Hospital of Harbin Medical University, China (IRB #2024XJSS10). All participants are required to sign written informed consent forms before participating in the studies.

## Supplementary Materials

### The PDF file includes:

Supplementary Text S1 to S5 Figs. S1 to S27 Tables S1 and S2 Legends for movies S1 to S4 References
