# Supplementary material for: Starfish-inspired wearable bioelectronic systems for physiological signal monitoring during motion and real-time heart disease diagnosis
Source: Sci Adv. 2025 Apr 2;11(14):eadv2406. doi: 10.1126/sciadv.adv2406 (PMC11963991; doi:10.1126/sciadv.adv2406)
Supplement: Supplementary file 1 — Supplementary Text S1 to S5 Figs. S1 to S27 Tables S1 and S2 Legends for movies S1 to S4 References [file sciadv.adv2406_sm.pdf]

Science Advances
AAAS

Supplementary Materials for

Starfish-inspired wearable bioelectronic systems for physiological signal monitoring during motion and real-time heart disease diagnosis

Sicheng Chen *et al.*

Corresponding author: Zheng Yan, yanzheng@missouri.edu; Sicheng Chen, scxmn@missouri.edu

Sci. Adv. **11**, eadv2406 (2025)
DOI: 10.1126/sciadv.adv2406

The PDF file includes:

Supplementary Text S1 to S5
Figs. S1 to S27
Tables S1 and S2
Legends for movies S1 to S4
References

Other Supplementary Material for this manuscript includes the following:

Movies S1 to S4

Supplementary Text

Supplementary Text S1

The stress coupling coefficient (α_c) is defined as the ratio of the average stress along the diagonal axes (S_1) to the average stress on the arm actively undergoing displacement (S_2).

$$\alpha_c = \frac{S_1}{S_2} = \frac{\iint \sigma_1\, d(A_1)}{\iint d(A_1)} \times \frac{\iint d(A_2)}{\iint \sigma_2\, d(A_2)}$$

Here, $\sigma_1$and A_1 represent the stress and area along the diagonal axes, while σ_2 and A_2 represent the stress and area on the axes undergoing displacement.

We can further use the α_c and the Signal-to-Noise Ratio (SNR) to quantify how much a signal is affected by noise. It is expressed in decibels (dB) and represents the ratio between the power of the desired signal (useful information) and the power of the background noise (unwanted interference). A higher SNR indicates a clearer and more distinguishable signal, with the signal being much stronger than the noise, leading to improved clarity and quality (*42*). Conversely, a low SNR suggests that the noise level is high relative to the signal, which can degrade the quality or intelligibility of the transmitted or received information.

During dynamic simulations, we can determine the signal power (K) from real-time displacement (A). To simplify the process, we assume that the energy input comes from motor vibration and consider the process as simple harmonic motion. The velocity of each arm tip (V) is the first derivative of displacement with respect to time:

$$V = \frac{dA(t)}{dt} = -A\omega sin(\omega t + \varphi)$$

From this, the signal power can be expressed as:

$$K = \frac{1}{2}\mathrm{m}(-A\omega sin(\omega t + \varphi))^2 = \frac{1}{2}\mathrm{m}A^2\omega^2 sin^2(\omega t + \varphi)$$

Without considering the effects of phase differences and Doppler shifts, the SNR can be approximately calculated as:

$$SNR = 10log_{10}(\frac{K_S}{K_N}) = 10log_{10}(\frac{\frac{1}{2}\mathrm{m}{A_S}^2\omega^2 sin^2(\omega t + \varphi)}{\frac{1}{2}\mathrm{m}{A_N}^2\omega^2 sin^2(\omega t + \varphi)})$$

For multi-arm configurations, we set the background noise (e.g. motion artifacts) as N and the target signal as S in a single arm. The composited target arm should have an output of S+N. The coupling arm has an output of α_cS+N, where α_c is the coupling coefficient. Here, we firstly normalize the signal before processing. So, the updated output on differential arm should be:

$$Outpout = \frac{\alpha_C \mathrm{S} + \mathrm{N}}{{\alpha_C}^2 + 1^2}$$

After performing the differential operation, the output signal and updated relative SNR can be expressed as:

$$Signal = \frac{1}{1 + {\alpha_C}^2}S + \frac{{\alpha_C}^2}{1 + {\alpha_C}^2}N$$

$$relative\ SNR = 20log_{10}(\frac{1}{{\alpha_C}^2})$$

For the α_c = 15.7%, the relative SNR is 32.2 dB; for the α_c = 23.9%, the relative SNR is 24.9 dB; and for the α_c = 35.6%, the relative SNR is 17.9 dB.

Supplementary Text S2

ECG Waves: ECG signals capture the heart's electrical activity throughout the cardiac cycle, with each waveform corresponding to a specific physiological event. The P wave signifies atrial depolarization, the electrical activity that initiates atrial contraction and marks the beginning of the cardiac electrical cycle, signaling the atria's preparation to pump blood into the ventricles. The QRS complex reflects ventricular depolarization, the electrical activity that triggers ventricular contraction, which is the heart's primary pumping mechanism (*43*). Within the QRS complex, the Q wave is the initial negative deflection, the R wave is the subsequent positive deflection, and the S wave is any negative deflection following the R wave. This complex represents the activation of the ventricular muscles, leading to the contraction of ventricles and the pumping of blood to the body (from the left ventricle via the aorta) and the lungs (from the right ventricle via the pulmonary artery). The T wave signifies ventricular repolarization, marking the recovery phase as the ventricular muscles relax and return to a resting state (*44*).

GCG and SCG Waves: SCG and GCG signals reflect the mechanical aspects of the heart's diastolic and systolic phases, particularly the opening and closing of the aortic and mitral valves. Although both signals represent similar phases of cardiac activity, SCG reflects the heart's translational movement, while GCG represents its rotational movement, often displaying a phase difference (*45, 46*). Together, these signals provide a comprehensive understanding of cardiac mechanics. AC (Atrial Contraction), AO (Aortic Opening), MC (Mitral Closure), and MO (Mitral Opening) are key points marking different stages of cardiac activity, which can aid in the analysis of mechanical heart activity during the cardiac cycle (*47, 48*). AC corresponds to the atrial contraction phase, where the atria pump blood into the ventricles, and it aligns with the P wave on ECG (*49*). In echocardiography, the AC point marks the start of cardiac mechanical activity caused by atrial contraction (*50*). AO marks the opening of the aortic valve and the beginning of ventricular systole, as ventricular pressure exceeds aortic pressure, causing the valve to open and blood to flow into the aorta. MC represents the closure of the mitral valve during early ventricular systole, preventing blood from flowing back into the atria as ventricular pressure rises (*51*). MO indicates the mitral valve opening early in ventricular diastole, allowing blood to flow from the left atrium into the ventricle, preparing the heart for the next contraction (*52*).

G-Group Waves: In addition to marking the opening and closing of the aortic and mitral valves, GCG signals can be further categorized into distinct groups of g-waves. These include the g_I, g_J, g_K, and g_L waves (*53*), which correspond to specific events in the cardiac cycle. The g_I wave is characterized by a sharp downward notch on the y-axis, occurring around the ECG's R wave (*54*). The g_J wave, the most prominent peak on the y-axis, appears shortly after the R wave and and corresponds to the opening of the aortic valve (*55, 56*). The g_K wave appears as the initial notch in a lower-magnitude up-down wave during the mid-cardiac cycle, approximately after the ECG's T wave, and correlates with the second heart sound. Finally, the g_L wave is the subsequent notch in this up-down pattern, following the g_K wave.

Correlative information between heart mechanical and electrical signals: Pre-ejection period (PEP), left ventricular ejection time (LVET), and electromechanical delay (EMD) represent the correlative information between cardiac mechanical and electrical signals. Their relationship with different motion states is detailed in supplementary Text S5.

Supplementary Text S3

While BLE transmission can involve occasional packet loss, we have implemented two methods to mitigate its impact on signal quality and ensure data integrity. First, a data buffering mechanism is employed on the device to temporarily store data before transmission. This allows for retries in case of packet loss, ensuring that critical data is not lost. Additionally, BLE's built-in error correction protocols help identify and recover corrupted packets when possible. Second, to ensure abnormal waveforms are not caused by BLE transmission imperfections, the transmitted data is validated against the original signals stored on the device. For instance, on-device recordings are compared with data received on the mobile phone to confirm consistency and rule out transmission-induced artifacts.

To measure cardiac mechanical signals while preserving the flexibility of the starfish-inspired device, both accelerometers for SCG and gyroscopes for GCG are integrated into a single chip, the BMI270. The BMI270 is configured with an acceleration resolution of ±2 g at 16384 LSB/g and an angular velocity resolution of ±125 dps at 262.1 LSB/dps, with an acceleration noise level of 160 μg/√Hz and an angular velocity noise level of 0.008 dps/√Hz. It supports a maximum acceleration output rate of 1.6 kHz and a maximum angular velocity output rate of 6.4 kHz. By using the clock signal from the nRF52-series processor to trigger all interfaces simultaneously at a consistent 200 Hz frequency, the device ensures a stable and reliable sampling rate/output data rate across all channels.

When the starfish-inspired device is affixed to the chest, the x-axis of the BMI270 aligns from the left hand to the right hand, the y-axis from the head to the feet, and the z-axis from the back to the front. In addition to capturing cardiac mechanical signals, the BMI270 can simultaneously monitor human motion and physical activity. The recorded data is processed using our machine-learning models to achieve high-fidelity SCG and GCG recordings even during motion (Fig. 3 and Fig. 4).

Supplementary Text S4

The accelerometer and gyroscope on Arm 5, positioned closest to the heart, collect signals composed of both cardiac mechanical signals (f_{cms}) and motion-induced mechanical signals (f_{mms}) during motion. The f_{mms} component can be inferred based on the real-time motion states of the other four arms (i.e., Arms 1-4), which do not contain strong cardiac mechanical signals (Fig. 2B), as achieved through the synthesis of real-time motion vectors from these arms, as shown in Fig. S8A(i). By simplifying and eliminating the coordinates of the central point, we can derive the final transformation relationship as:

$$\overrightarrow{A_4A_1} + \overrightarrow{A_4A_2} = \mathrm{k}(\overrightarrow{A_4A_3} + \overrightarrow{A_4A_5}).$$

The resultant vector formed by vectors A_4A_5 and A_4A_3, as shown in fig. S8A (ii), is directed along the axis of symmetry. The magnitude of this resultant vector is:

$$L1 = \cos 54^{\circ}\left(\sqrt{(x_4 - x_5)^2 + (y_4 - y_5)^2 + (z_4 - z_5)^2} + \sqrt{(x_4 - x_3)^2 + (y_4 - y_3)^2 + (z_4 - z_3)^2}\right)$$

Similarly, in fig. S8A (iii), the resultant vector formed by vectors A_4A_1 and A_4A_2 is also directed along the axis of symmetry with a magnitude of:

$$L2 = \cos 16^{\circ}\left(\sqrt{(x_4 - x_1)^2 + (y_4 - y_1)^2 + (z_4 - z_1)^2} + \sqrt{(x_4 - x_2)^2 + (y_4 - y_2)^2 + (z_4 - z_2)^2}\right)$$

Assuming the structure forms a perfectly symmetrical pentagon, we can calculate:

$$k = \frac{L1}{L2} = \frac{2\cos 54^{\circ}}{2\cos 16^{\circ} \times 2\cos 38^{\circ}} = 0.388$$

Thus, we can express the relationship as $\overrightarrow{A_4A_1} + \overrightarrow{A_4A_2} = 0.388(\overrightarrow{A_4A_3} + \overrightarrow{A_4A_5})$.

By subtracting this f_{mms} component from the signal collected by Arm 5 (f_{cms} + f_{mms}), we can extract the raw cardiac mechanical signal (f_{cms}). The flowchart depicting this entire process is shown in fig. S8B. In practice, the value of k might require slight adjustments due to potential processing inaccuracies in the starfish-like device and deviations that occur when affixed to non-perfectly flat surfaces. Despite these variables, the construction process and computational approach remain consistent with the described methodology.

Supplementary Text S5

Electromechanical delay (EMD) is the time interval between the onset of the heart's electrical signal, typically marked by the beginning of the QRS complex on the ECG, and the initiation of mechanical contraction in the heart muscle, commonly measured as the S1 peak in heart mechanical signals (*57*). This delay occurs because after the electrical system signals the heart to contract, there is a brief period before the electrical stimulus triggers the muscle fibers to contract and generate a mechanical response (*58*). EMD can provide insights into the synchronization between the heart's electrical and mechanical functions. A prolonged EMD may indicate impaired electromechanical coupling, often associated with cardiac dysfunctions such as heart failure or conduction abnormalities (*59, 60*).

Pre-ejection period (PEP) refers to the time from the onset of ventricular depolarization, marked by the Q wave on the ECG (*61*), to the opening of the aortic valve (AO, typically measured in cardiac mechanical signals). It consists of two phases: isovolumetric contraction (when the ventricles are contracting but not yet ejecting blood) and the electromechanical delay. PEP reflects the time required for the ventricles to generate sufficient pressure to open the aortic valve and begin blood ejection. A prolonged PEP can indicate delayed or weakened ventricular contraction, commonly seen in conditions such as heart failure or reduced myocardial contractility. Conversely, a shortened PEP may be observed in hyperdynamic states, such as during physical exercise or in conditions like hyperthyroidism (*62*).

Left ventricular ejection time (LVET) measures the duration of blood ejection from the left ventricle into the aorta, beginning with the opening of the aortic valve (AO) and ending with its closure (AC), as measured in cardiac mechanical signals (*63*). LVET provides direct insight into the heart's pumping efficiency. A reduced LVET can indicate compromised cardiac output, as seen in heart failure or aortic stenosis (*64*), while an extended LVET may occur in hyperdynamic states or when ventricular emptying is prolonged due to decreased afterload, as in certain cases of valvular insufficiency (*65*). The PEP/LVET ratio is a valuable index for assessing cardiac contractility and overall heart function. A lower ratio typically indicates better cardiac performance and efficiency.

Together, these parameters offer a deeper understanding of the relationship between the electrical signals that initiate heart contractions and the subsequent mechanical actions. Variations in these parameters can reveal cardiac pathologies or altered physiological states, offering valuable insights for diagnosing and monitoring heart conditions.

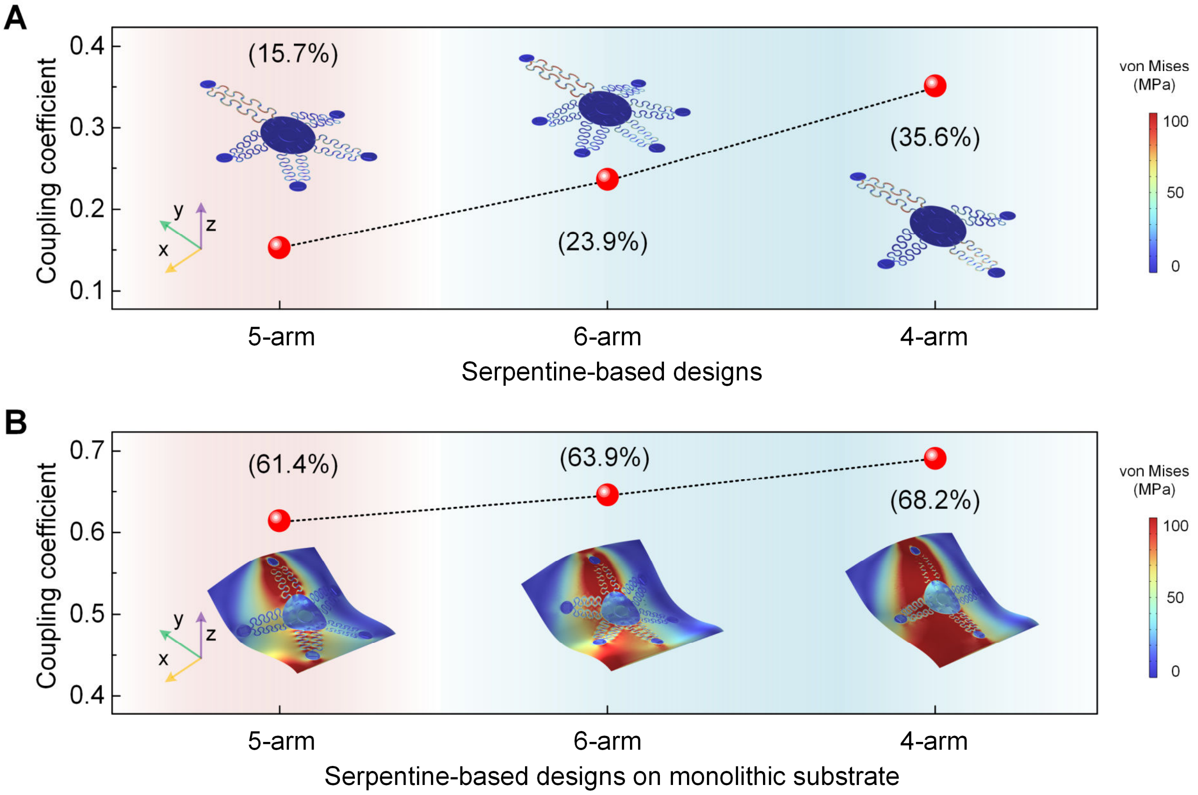

Fig. S1. Superiority of the starfish-inspired five-arm configuration compared to other serpentine-based designs and the monolithic form design. (A) The five-arm design achieves the lowest stress coupling coefficient at 15.7%, significantly lower than those of the four-arm (35.6%) and six-arm (23.9%) configurations, despite all being serpentine-based designs. This highlights the advantages of the starfish-like form factor over other serpentine-based structures. **(B)** Incorporating a monolithic substrate into these serpentine-based designs substantially increases the device's coupling coefficient to 61.4% (five-arm), 63.9% (six-arm), and 68.2% (four-arm), further reinforcing the superiority of the starfish-inspired configuration over monolithic designs.

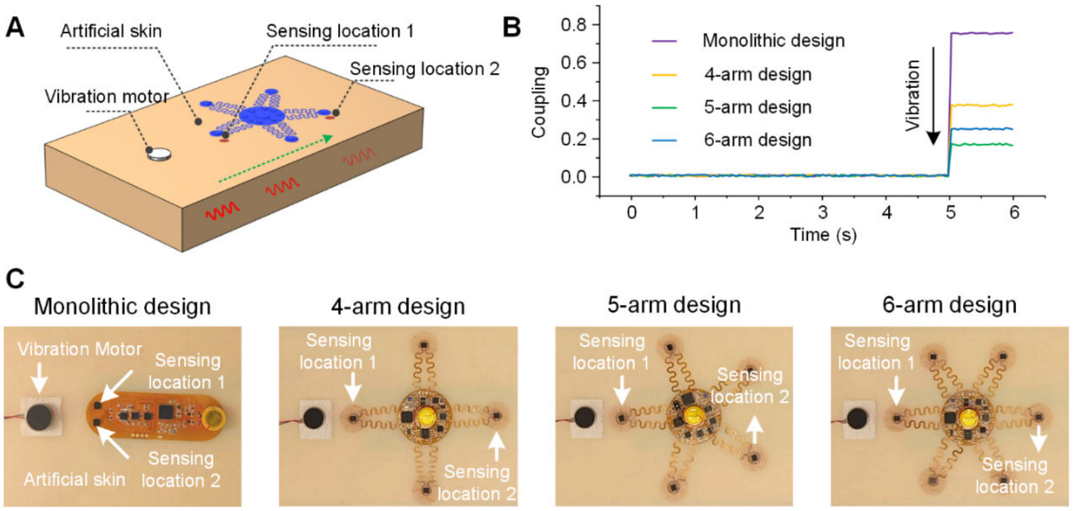

Fig. S2. Mechanical vibration experiments with various device configurations. (A) Experimental setup with the device placed on artificial skin and a vibration motor positioned at one end. The motor is controlled by voltage toggling, and displacement curves of each sensing element are collected during operation. These displacement values are converted into correlation coefficients (ratios of displacement at location 2 to displacement at location 1) to evaluate mechanical coupling. **(B)** Comparative analysis of mechanical coupling across different device configurations: traditional monolithic design, and four-arm, five-arm, and six-arm radial symmetries. The results show that the five-arm configuration significantly reduces mechanical coupling compared to the other designs. **(C)** Images of the various device configurations placed on artificial skin for the mechanical vibration tests.

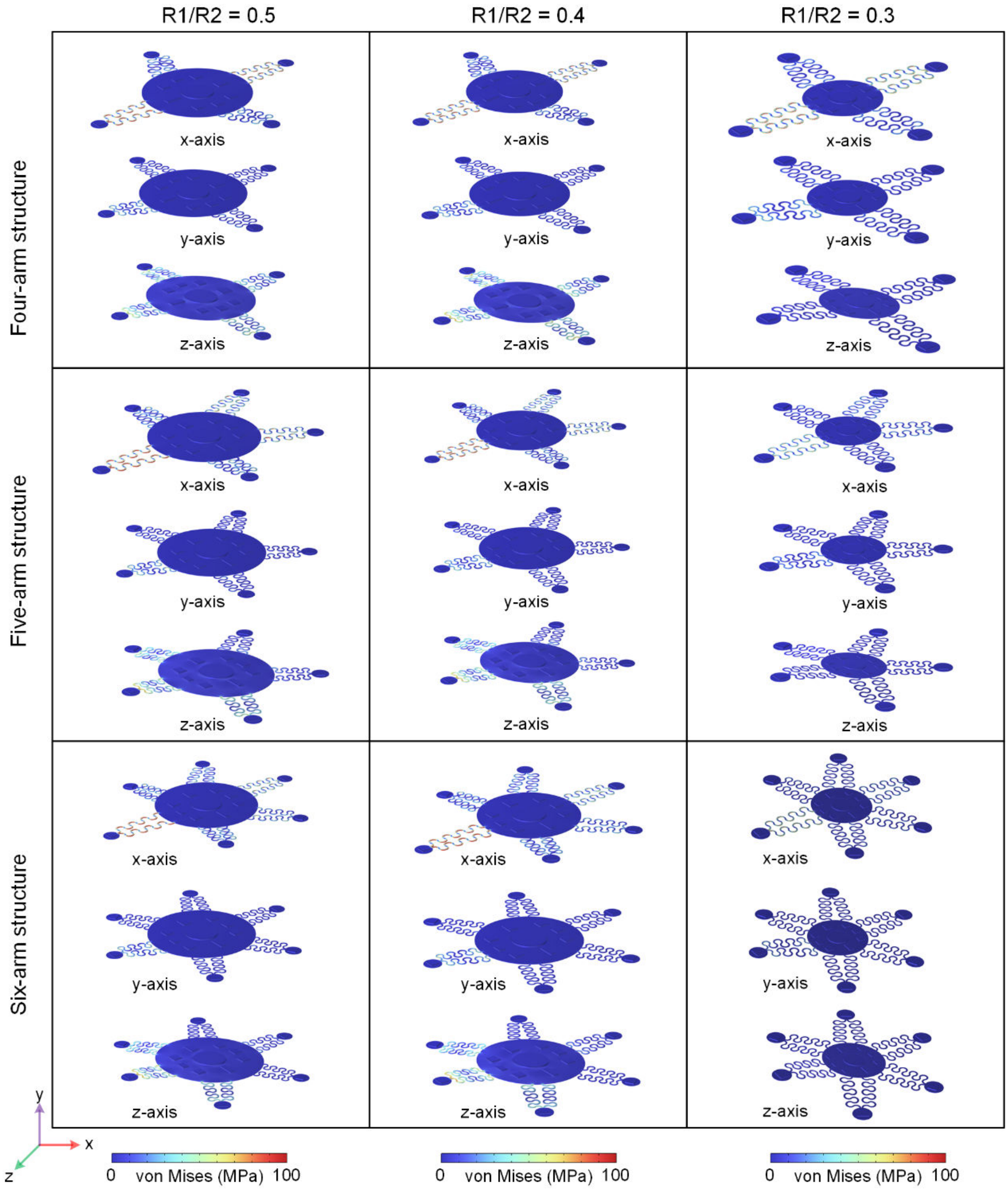

Fig. S3. Coupling conditions of various arms configurations and R1/R2 ratios. Each column Each model simulates the coupling behavior in three dimensions (x, y, z) for device designs with 4-arm, 5-arm, and 6-arm configurations. Mechanical coupling capabilities are evaluated and converted into SNR parameters for comparative analysis (Fig. 1C). The simulations are performed for R1/R2 ratios of 0.5, 0.4, and 0.3, as shown in three columns. The results demonstrate that the five-arm configuration consistently achieves the highest SNR across all R1/R2 ratios, indicating superior performance in reducing mechanical coupling compared to the four-arm and six-arm configurations.

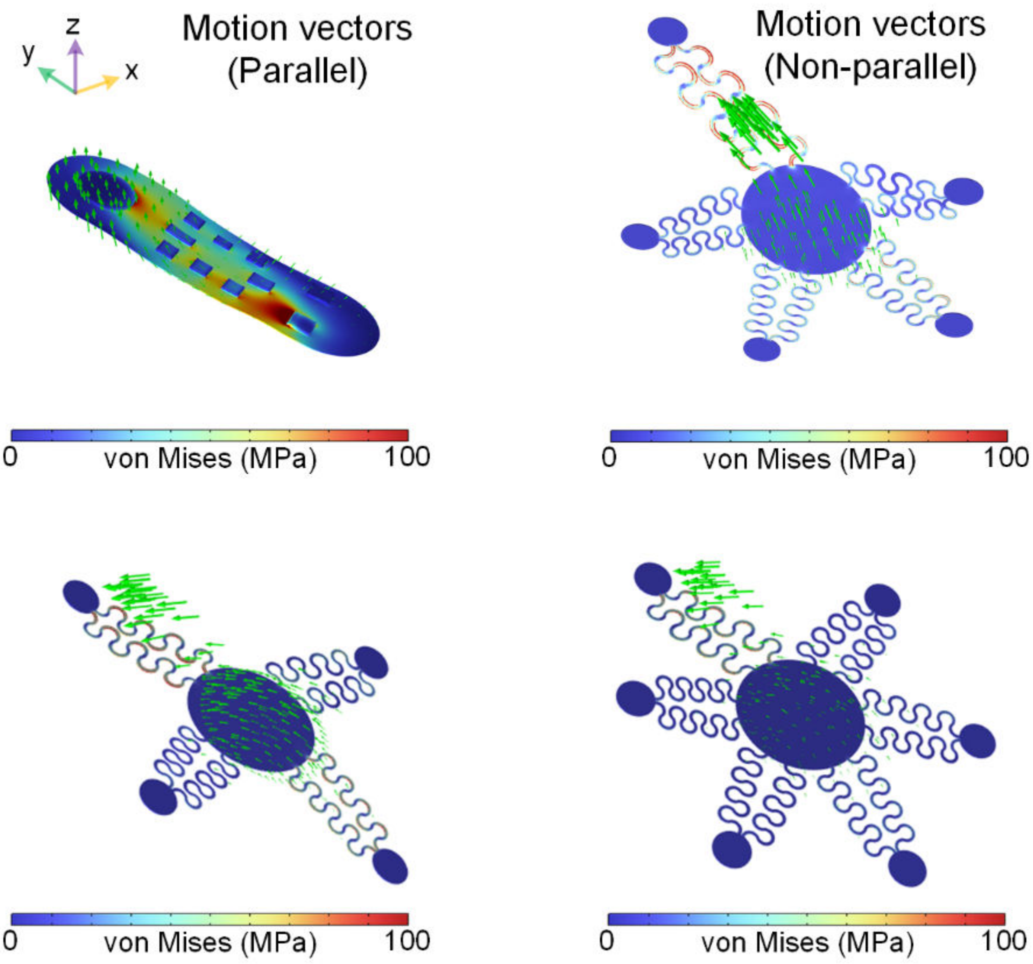

Fig. S4. Displacement vectors of monolithic and four-, five- and six-arm starfish-like designs under mechanical stimuli. Displacement vectors of a monolithic device and four-, five- and six-arm starfish-like configurations when subjected to mechanical stimuli, illustrating how each part of the device moves in response to external forces. In the monolithic design, displacement vectors tend to be parallel, indicating restricted movement, predominantly perpendicular to the substrate surface. This behavior limits the device's flexibility and adaptability under dynamic conditions. In contrast, the five-arm configuration demonstrates non-parallel displacement vectors, allowing for more diverse and flexible movement. This increased flexibility enables sensing elements to better adapt to varying mechanical forces, reducing mechanical coupling and improving the accuracy of biosignal collection. Although the four-arm and six-arm configurations offer greater flexibility compared to the monolithic design, they are outperformed by the five-arm configuration.

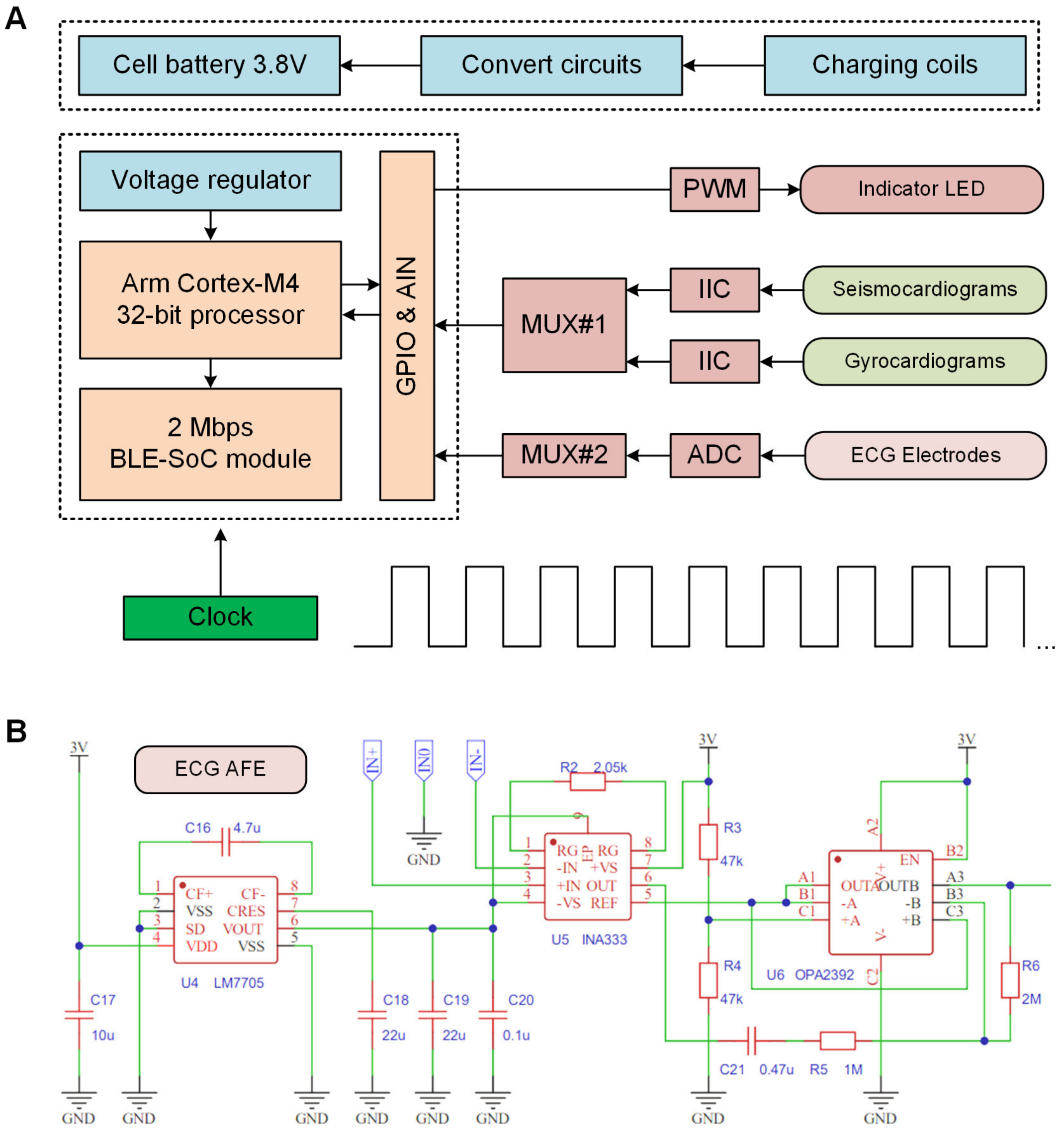

Fig. S5. System architecture of the starfish-like device and ECG AFE circuits. (A) The core components of the starfish-like device include the energy supply module, processor module, and data acquisition module. ECG sensors, accelerometers, and gyroscopes are connected to the data acquisition circuit. The system is powered by an onboard cell battery, supplemented by wireless charging coils. Accelerometers and gyroscopes generate high-frequency digital signals, which are fed into a multiplexer (MUX) through an IIC interface and communicated to the 32-bit processor via general-purpose input/output (GPIO) ports. Simultaneously, analog signals from the ECG electrodes are digitized by an analog-to-digital converter (ADC) and routed through another MUX to the processor's analog input (AIN) ports, following the same timing sequence. After processing the signals recorded by ECG sensors, accelerometers, and gyroscopes using on-chip algorithms, the processor transmits the data to an external mobile device via a high-speed 2 Mbps Bluetooth system-on-chip (SoC). **(B)** The ECG analog front-end (AFE) circuit employs a multi-stage design with adjustable gain and filtering capabilities, consisting of three main stages: negative voltage generation, multi-stage amplification, and filtering.

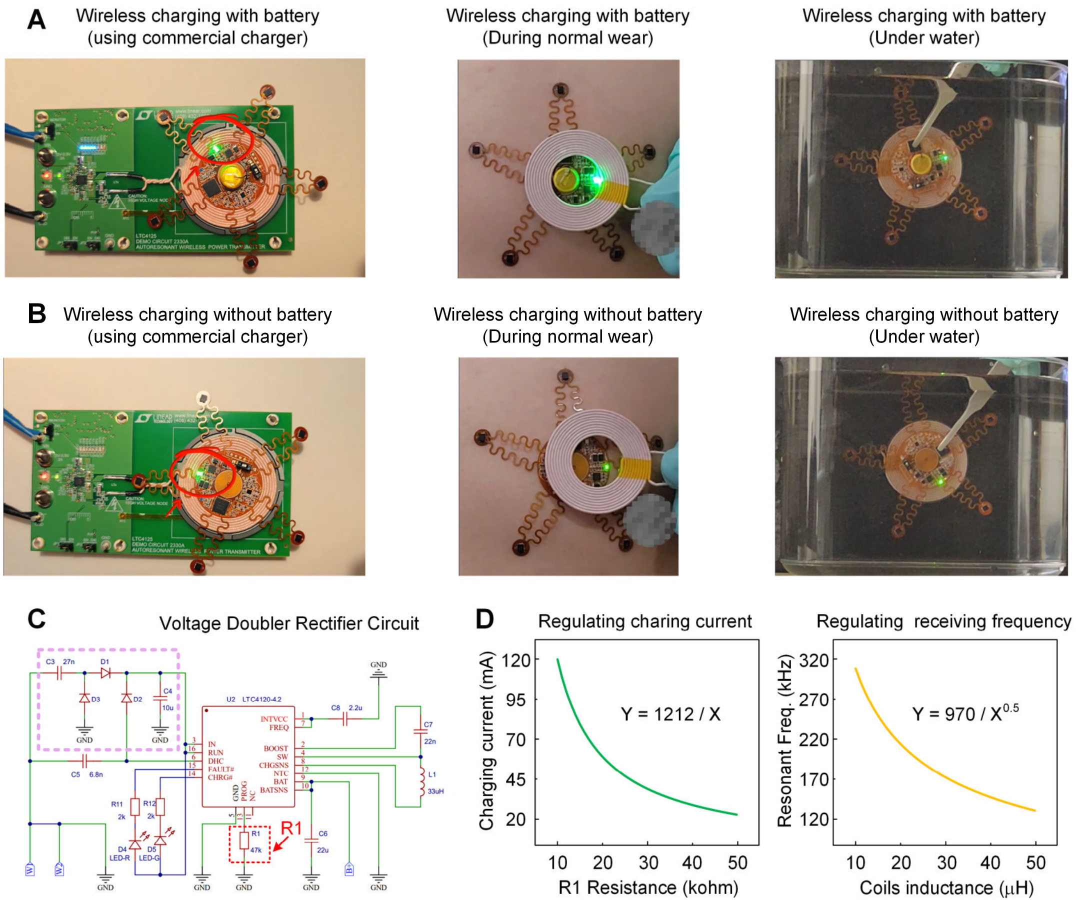

Fig. S6. Wireless recharging and related parameters. (A) Wireless recharging of the starfish-like device with battery under various conditions. The device can be wirelessly recharged using a commercial charger, on human skin, and even underwater. It charges efficiently in close proximity to commercial wireless chargers or by harvesting energy from nearby wireless charging coil during operation, ensuring continuous, long-lasting functionality without the need for battery replacement. Additionally, the device can harvest energy from ambient electromagnetic fields in underwater environments and simulated sweat conditions, enabling uninterrupted signal collection during various daily activities. **(B)** Wireless powering of the starfish-like device with a battery under various conditions. The device can be wirelessly powered using a commercial charger, on human skin, and even underwater. In this scenario, magnetic field energy is directly converted into electrical energy to supply the system, allowing for normal operation without relying on the battery. **(C)** Wireless charging circuit with regulated charging current and receiving frequency. The wireless charging process consists of two main stages: voltage input through the receiving coil, followed by rectification. On the coil area of the starfish-like device, a voltage doubler circuit is employed to boost the input voltage. **(D)** Charging current regulation and receiving frequency adjustment. The charging current is inversely proportional to the resistance of R1, meaning that as resistance increases, the charging current decreases, with their product remaining constant at 1212 kΩ·mA. Similarly, the frequency of wireless charging is inversely proportional to the inductance of the receiving coil, with their product being 970 kHz·μH. As the coil's inductance increases, the resonance frequency decreases accordingly.

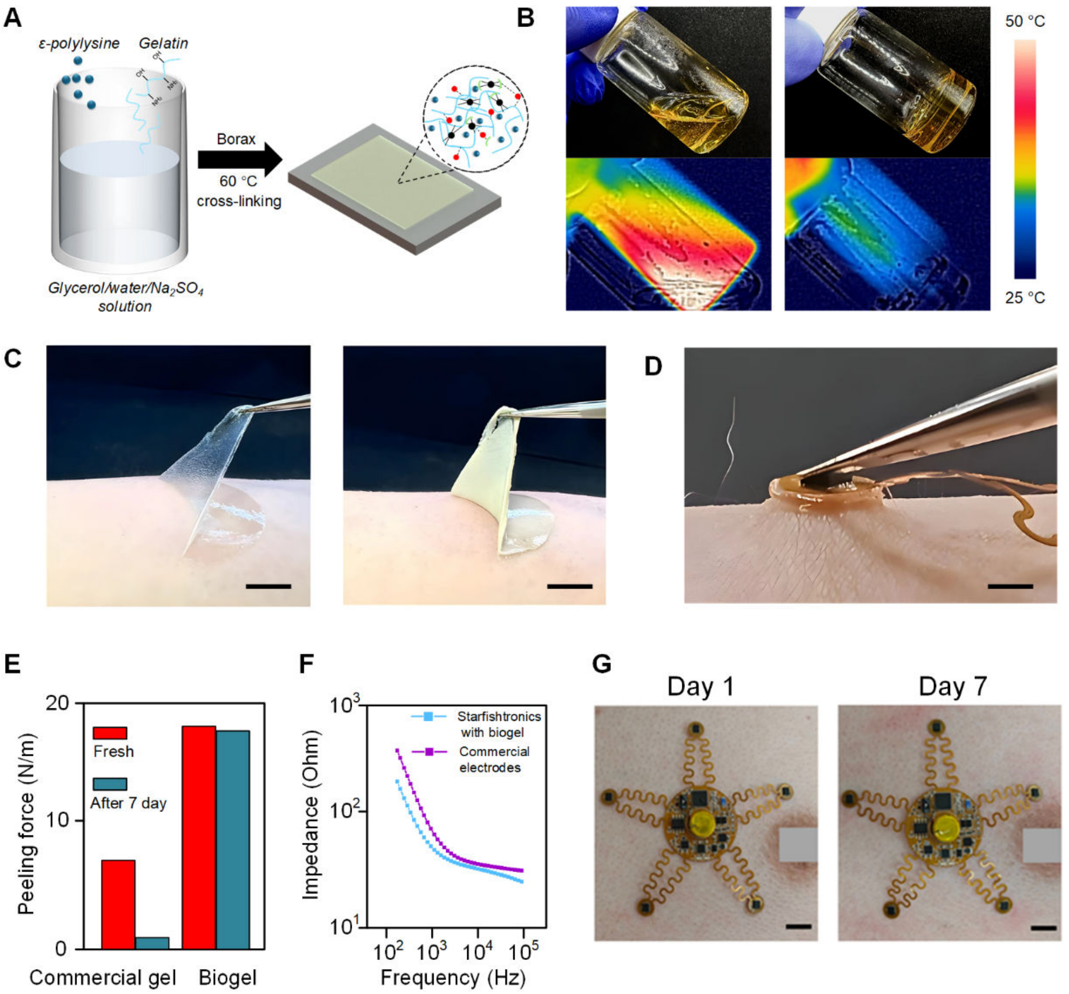

Fig. S7. Long-term stable adhesive biogel for the starfish-like device-skin interfacing. (A) Schematic illustration of the adhesive biogel synthesis (see Materials and Methods for the details). **(B)** Optical images of the biogel in different states: fluid at 50°C (left) and solid-like at 25°C (right). **(C)** Optical images showing the peeling of the biogel (left) and conductive biogel (right, biogel mixed with silver nanowires) from human skin, indicating strong adhesive contact. Scale bars: 2 cm. **(D)** Optical image, showing robust adhesion of the sensing pad to human skin, facilitated by the conductive biogel. The image features a tweezer used to stretch the sensing pad from the skin. Scale bar: 0.3 cm. **(E)** Comparison of 90-degree peeling forces between commercial gel and the biogel on Day 1 and Day 7, demonstrating the long-term stability of the biogel. **(F)** Device-skin impedance comparison between commercial silver/silver chloride (Ag/AgCl) gel electrodes (3M 2560-5 Red Dot Monitoring Electrode) and the starfish-like device coated with conductive biogel, showing significantly reduced impedance, which can enhance ECG recording quality. **(G)** Optical images of the starfish-like device attached to human chest skin on Day 1 and Day 7 using the biogel. Scale bars: 1.4 cm.

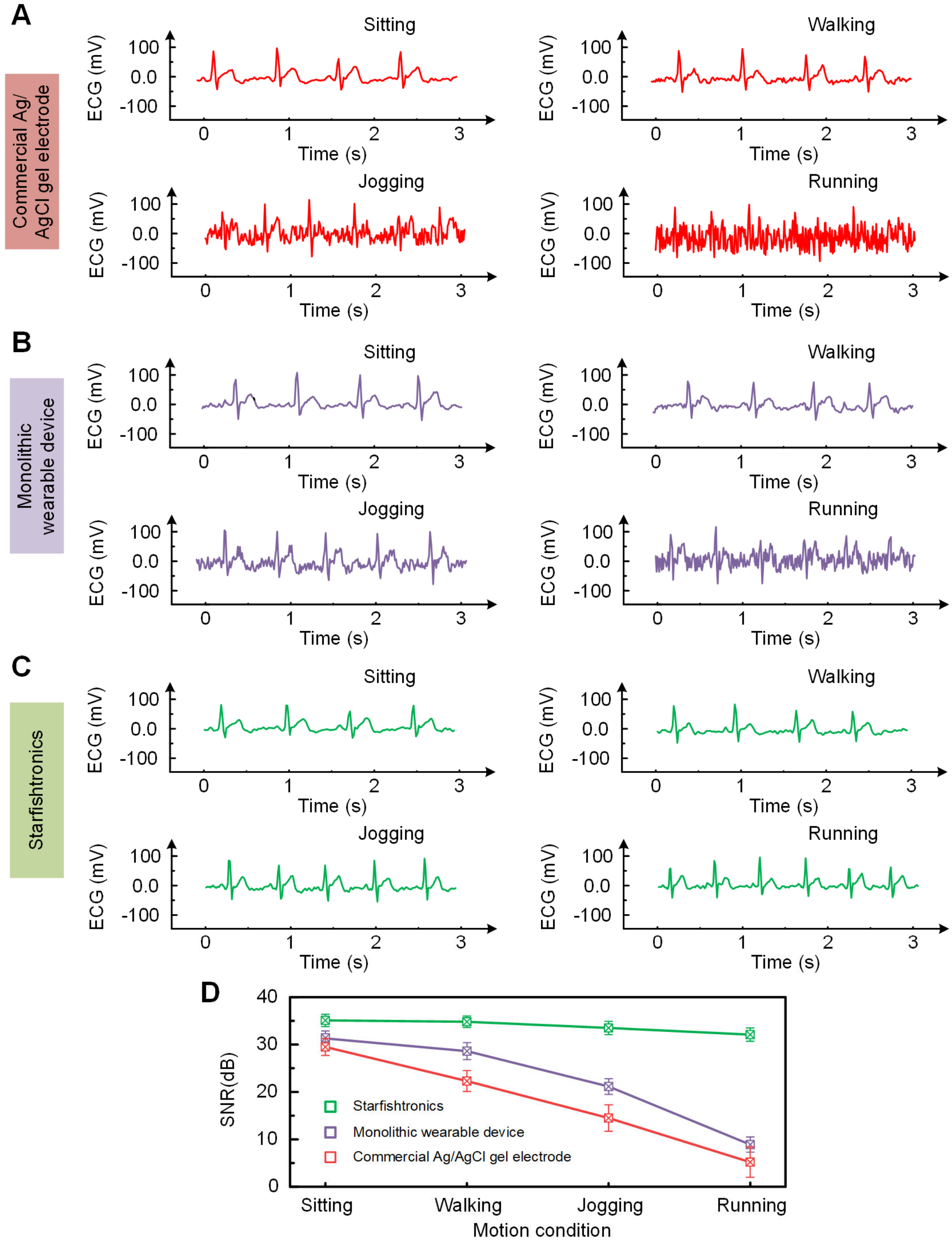

Fig. S8. Comparison of ECG signals recorded using various devices. ECG signals recorded from human subjects across different motion states (sitting, walking, jogging, and running) using **(A)** commercial Ag/AgCl gel electrodes, **(B)** traditional monolithic wearable devices, and **(C)** the starfish-like device interfaced with human skin using biogel. **(D)** SNR of ECG signals collected across different motion states with various devices, showing that the starfish-like device provides high-fidelity and stable ECG recordings during motion. This performance is attributed to the robust biogel interface and mechanically decoupled starfish-like device configuration.

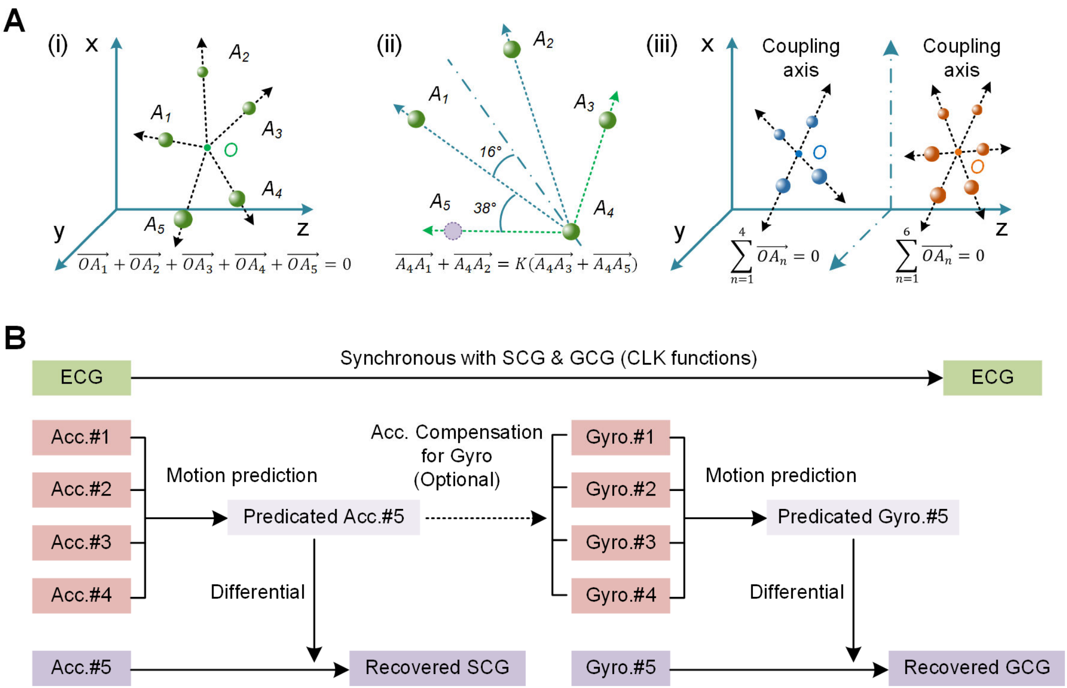

Fig. S9. Motion prediction for Arm 5 and flowchart of the overall process. (A) The motion-induced mechanical signals of (i) all arms can be inferred based on the real-time motion states of (ii) the other four reference arms through vector synthesis. (iii) In addition to the 5-arm design, both the 4-arm and 6-arm configurations can also achieve vector synthesis in a similar manner, indicating the universality of this approach. **(B)** The acceleration and rotational movements of any arm can be predicted through vector synthesis using motion data from the other four arms. By isolating and removing the motion signals from Arm 5, the cardiac mechanical signals are obtained. In this process, Acc. #1-5 and Gyro #1-5 represent the accelerometers and gyroscopes embedded in sensing pads 1-5. The CLK function refers to the clock signal used to synchronize data acquisition and processing across all sensors, ensuring simultaneous capture and consistent alignment of ECG, SCG, and GCG data. This synchronization is crucial for accurate motion compensation and precise cardiac signal analysis.

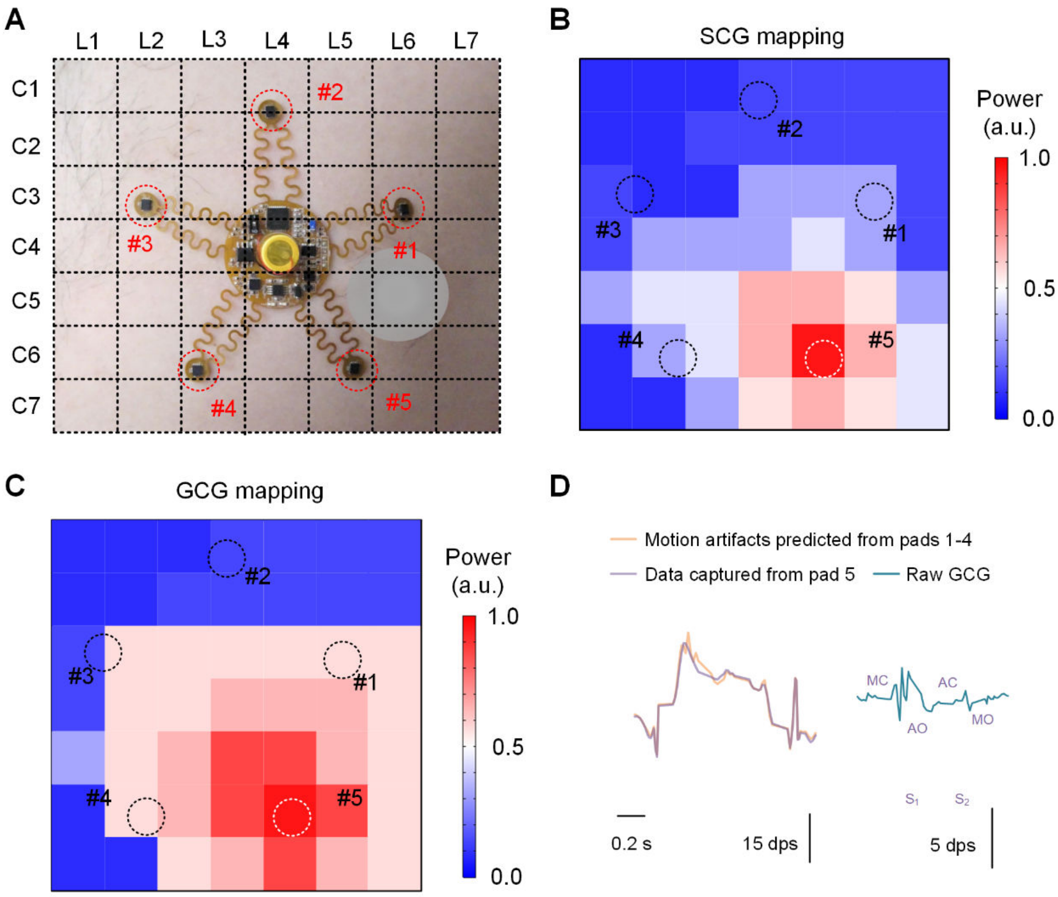

Fig. S10. Power density mapping of SCG and CGG signals on the human chest and corresponding GCG waves. (A) The skin area near the heart is divided into a 7x7 grid of 49 regions, each tested using a single sensing arm of the starfish-like device to generate a comprehensive mapping of cardiac mechanical signals. The intensity data is normalized and color-coded to visualize the distribution across the entire area. **(B)** SCG mapping, showing acceleration energy distribution, with the highest energy detected at sensing pad 5. **(C)** GCG mapping, displaying a generally positive correlation with SCG results. The GCG energy trend is most pronounced near the heart's apex. **(D)** Motion artifacts (orange) on sensing pad 5, derived from the motion signals recorded by sensing pads 1-4, alongside signals (purple) containing both motion artifacts and GCG signals captured by the gyroscope on sensing pad 5. The raw GCG signal (blue) on sensing pad 5 is the differential result of the two profiles (orange and purple).

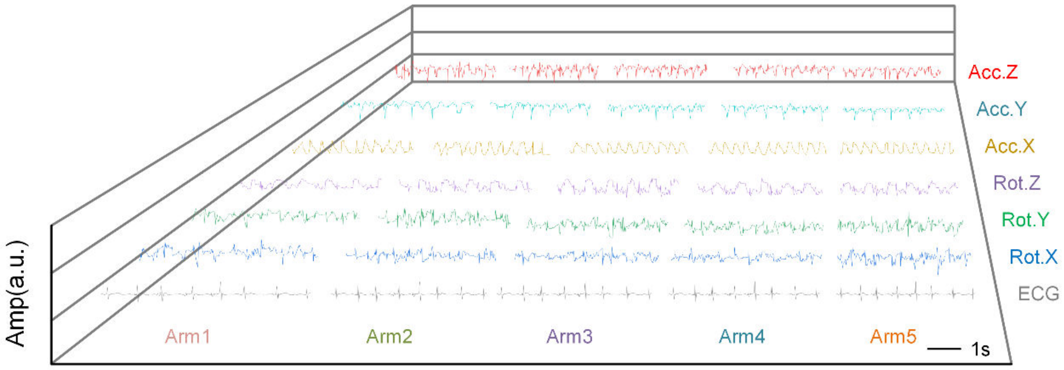

Fig. S11. Raw data collected from all recording channels using the starfish-like device. The dataset includes mechanical signals from 30 channels and ECG signals from five channels. Each arm's accelerometer captures three channels of acceleration signals, while each gyroscope captures three channels of rotational signals. During running, the amplitude of mechanical signals increases significantly in all dimensions, with the Z-direction acceleration amplitude rising over tenfold. Additionally, the signals exhibit rhythmic, stride-synchronized periodic patterns. Rhythmic beats are observed in both the rotational and translational signals from each arm, primarily reflecting coordinated body movements. Despite these mechanical variations, the electrical signals maintain a stable signal-to-noise ratio. Using ECG signals as a reference, it is possible to anchor the mechanical signals for subsequent analysis of the relationship between mechanical and electrical signals.

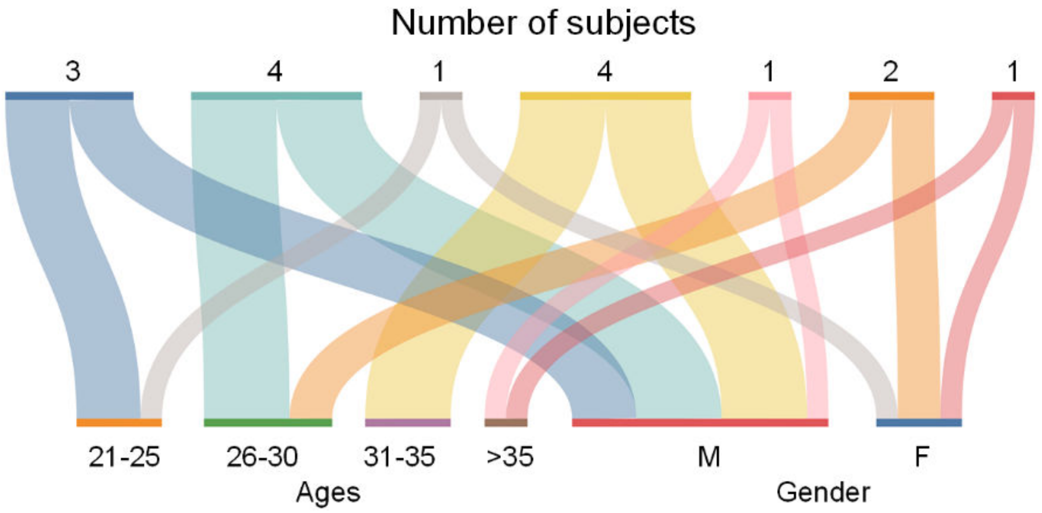

Fig. S12. Distribution of participant demographics for data collection. Data were collected from 16 volunteers under various physical states for model training. Participants ranged in age from 21 to over 35 years, including both male and female individuals. The age distribution includes four participants aged 21-25, six participants aged 26-30, four participants aged 31-35, and two participants over 35. The sample set comprised 12 male and 4 female participants.

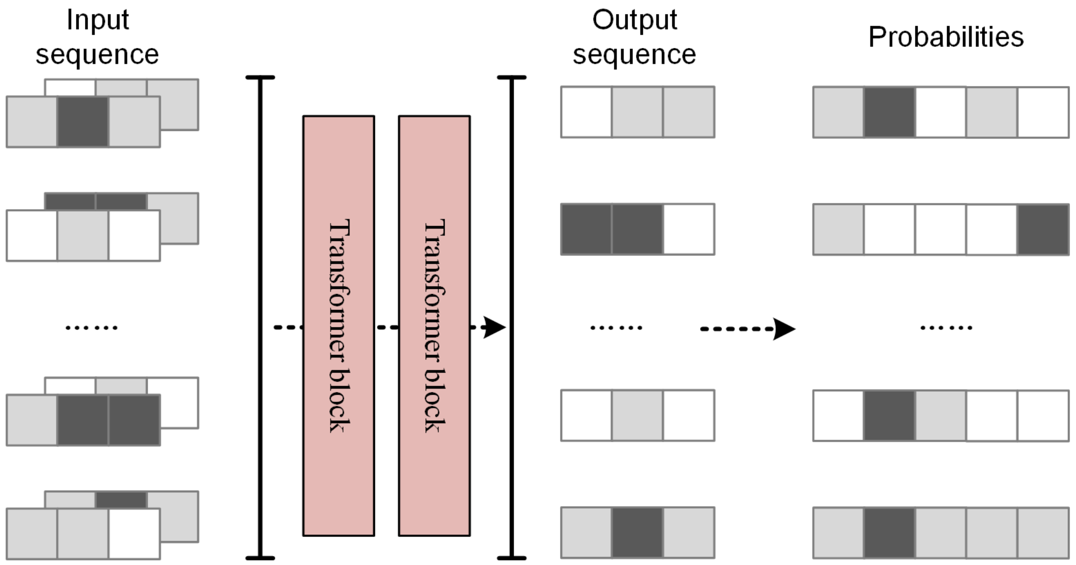

Fig. S13. Signal processing workflow using the transformer model. The signal at each timestep t corresponds to a token in the transformer encoding block at timestep t. The transformer outputs motion state predictions for each timestep. By incorporating temporal information within each sliding window, the transformer enhances prediction accuracy by leveraging contextual data. Other machine learning models used in this work concatenate signals from all timesteps within the sliding window as a single input.

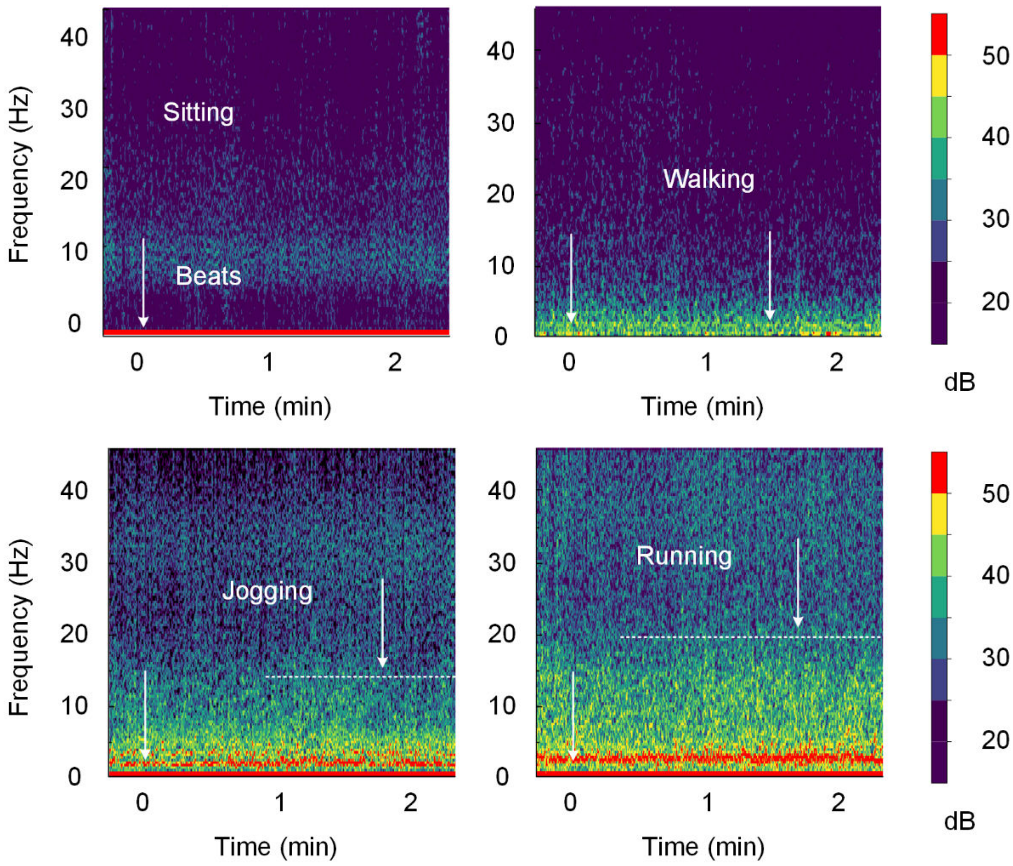

Fig. S14. Frequency domain distribution of mechanical signals under different motion states, analyzed using Short-Time Fourier Transform (STFT). The results reveal that the primary frequency range varies with motion states. As exercise intensity increases from sitting to running, there is a corresponding rise in the component of mechanical signals at higher frequencies, particularly ~20 Hz. To calculate the energy intensity (power spectral density) for a given frequency f and time t, the energy intensity is calculated as:

$$x = |\mathrm{Z(f,t)}|^2$$

where ∣ Z(f,t) ∣ is the magnitude of the Fourier coefficient, and squaring it gives the energy intensity at that frequency and time. For this analysis, we select the frequency range from 0 to 20 Hz and extract the energy intensity values across all time points, resulting in a matrix representing the energy intensity over time within the 0-20 Hz range. The mean energy intensity within this frequency range across all time points is calculated as:

$$\text{Average Energy Intensity} = \frac{1}{N}\sum_{f\in[0,20]}\sum_{t}|\mathrm{Z(f,t)}|^2$$

where N is the total number of data points in the 0-20 Hz frequency range, and $|\mathrm{Z(f,t)}|^2$ is the energy intensity at a specific frequency f and time t.

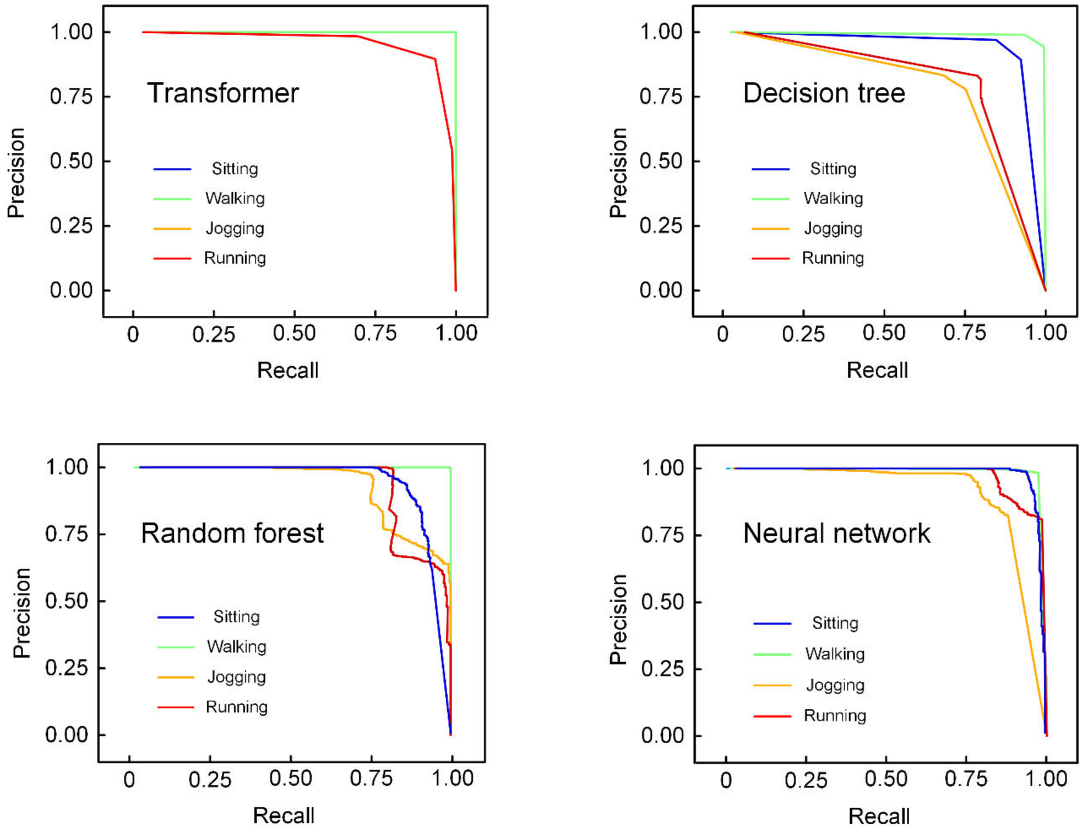

Fig. S15. Comparison of different machine learning models for motion recognition. The transformer model outperforms other models, including decision tree, random forest and neural network composed of fully-connected layers. Here, precision represents the proportion of positive identifications made by the model that are actually correct, where the Precision equals:

$$\text{Precision} = \frac{\text{TP}}{\text{TP} + \text{FP}}$$

TP (True Positive) refers to correct positive predictions, while FP (False Positive) refers to incorrect positive predictions. High precision means that the model is mostly correct when it predicts a positive result. Recall measures the proportion of actual positives that are correctly identified by the model. where the recall equals:

$$\text{Recall} = \frac{\text{TP}}{\text{TP} + \text{FN}}$$

FN (False Negative) represents actual positives that are predicted as negatives by the model. High recall indicates that the model is identifies most of the actual positives. Precision-Recall Curve displays the trade-off between precision and recall for different threshold values. Each point on the curve corresponds to a specific precision and recall value at a given threshold. By adjusting the model's decision threshold, the interplay between the precision and recall can be observed, with increasing precision typically leading to decreased recall, and vice versa.

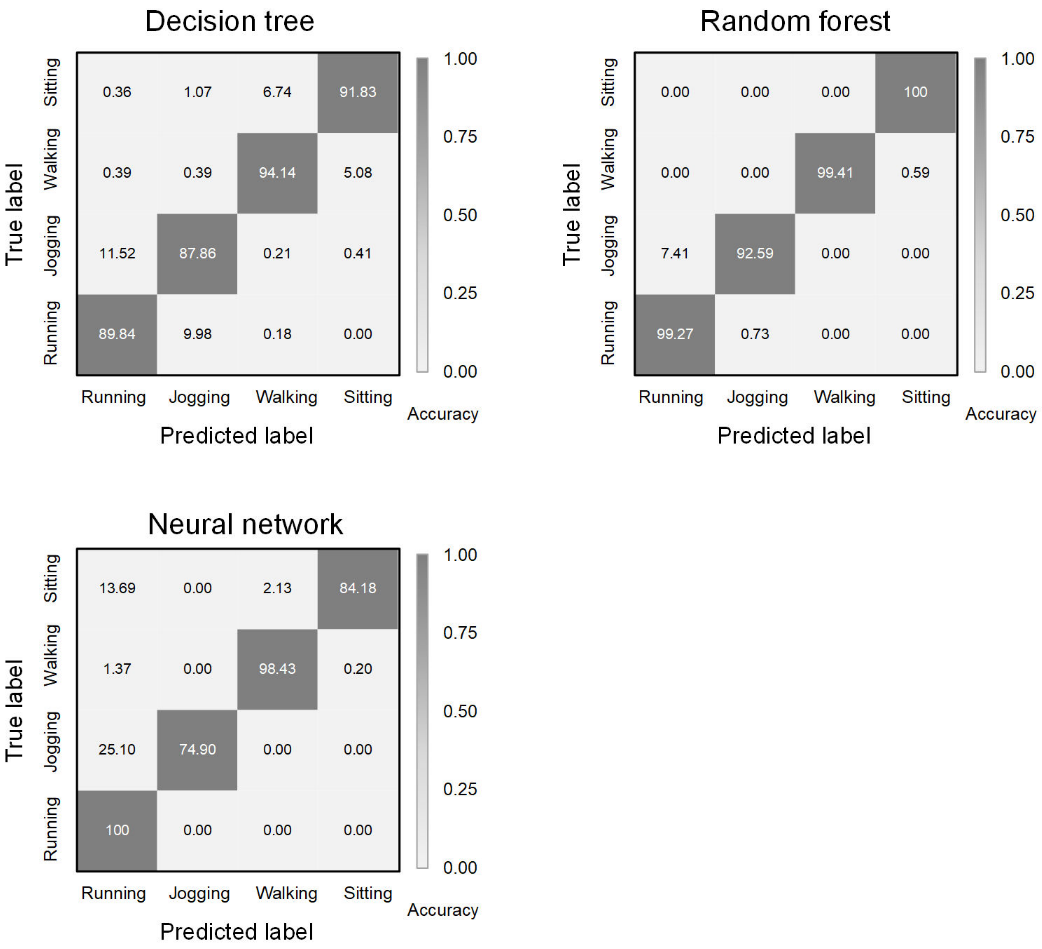

Fig. S16. Confusion matrix of different machine learning models displaying the classification accuracy for predicting each motion state in the test set. Combined with features extracted from both translational and rotational motion data, the Decision Tree model achieves accuracies of 91.83% for sitting, 94.14% for walking, 87.86% for jogging, and 89.84% for running. In comparison, the Random Forest model achieves accuracies of 100% for sitting, 99.41% for walking, 92.59% for jogging, and 99.27% for running. Meanwhile, the Neural Network model achieves accuracies of 84.18% for sitting, 98.43% for walking, 74.90% for jogging, and 100% for running.

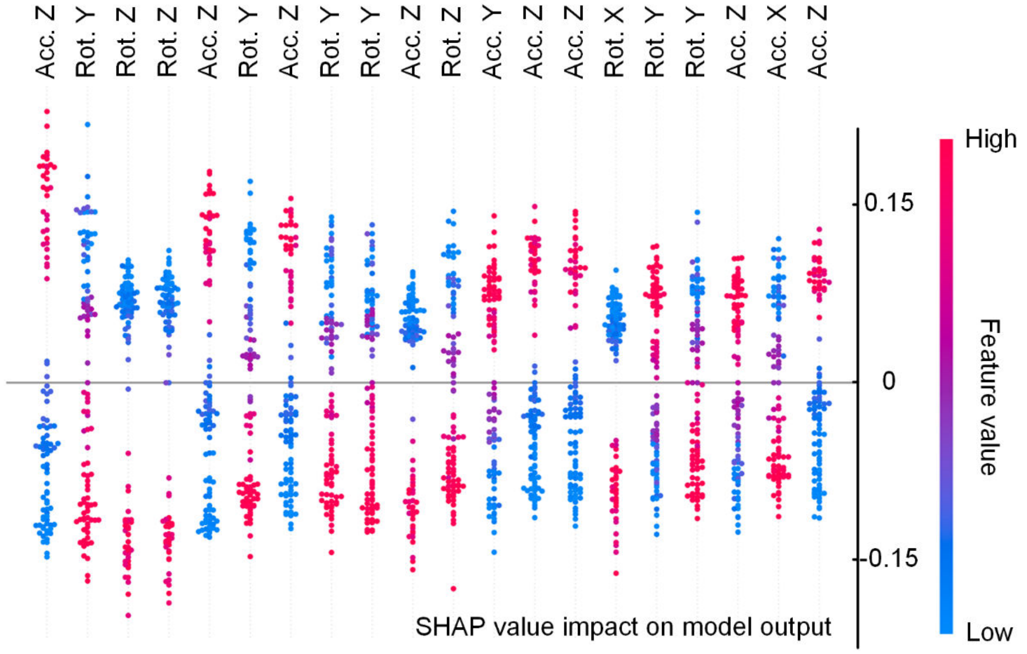

Fig. S17. SHAP summary plot for motion recognition. Through SHAP analysis, the feature importance of Acc. Z, Rot. Y, and Rot. Z indicates that these biosignals play a crucial role in motion recognition. This analysis calculates Shapley values for each feature to explain its impact on the model's predictions. SHAP values consider all possible combinations of features, providing a weighted decomposition of feature importance for each prediction. Since the model inputs in our study are not isolated data points but time series within a time window, multiple data points from a particular channel may appear repeatedly. Consequently, features within a time window can generate hundreds of feature values. We focus on the top ten features, considering them the most crucial determinants. Future model optimizations could leverage these key parameters to further simplify our machine-learning model and enhance its accuracy.

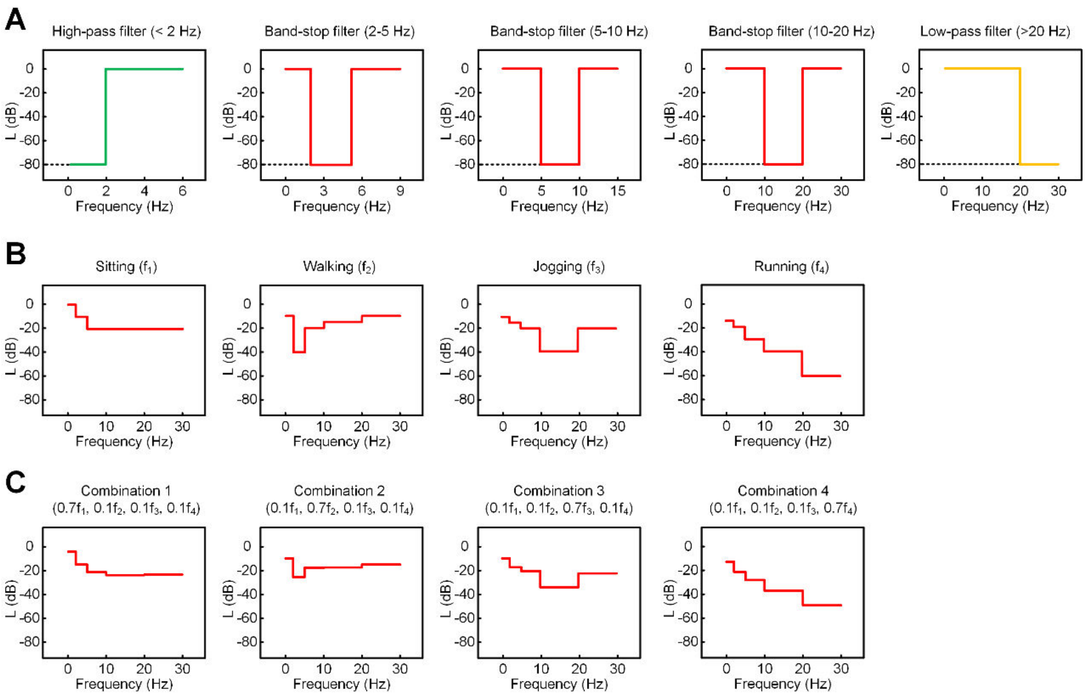

Fig. S18. Adaptive combination filtering for compensated cardiac biomechanical signals during various motion states. (**A**) Five basic filters designed for different frequency ranges: 0-2 Hz, 2-5 Hz, 5-10 Hz, 10-20 Hz, and above 20 Hz. (**B**) Adaptive filters for four motion states: sitting, walking, jogging, and running. Even when an actual motion state does not exactly fit within the four training motion states, the machine learning model outputs probabilities for all four states. This allows for the creation of a new combination filter based on the probability coefficients. (**C**) Four examples of these combinations represent different probabilities of the four main motion states of sitting, walking, jogging, and running. If we define the four adaptive filters as f_1, f_2, f_3, and f_4, and the combination coefficients as c_1, c_2, c_3, and c_4, then the real-time adaptive combination filter for a given motion state can be expressed as:

$$\text{Combination filter} = \sum_{i=1}^{4} f_i c_i$$

This equation shows how the combination filter integrates the effects of each adaptive filter, weighted by the corresponding coefficients for the ML-predicted motion state.

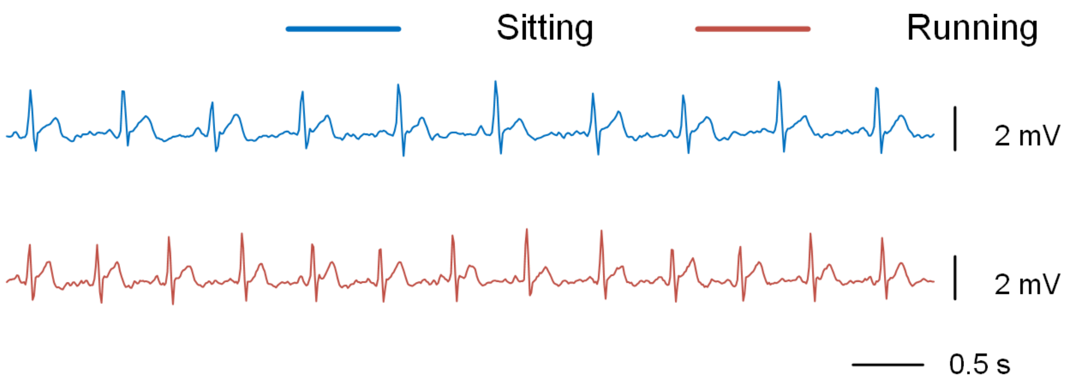

Fig. S19. ECG signals recorded from the same subject during sitting and running, concurrent with SCG and GCG signals shown in Fig. 4D.

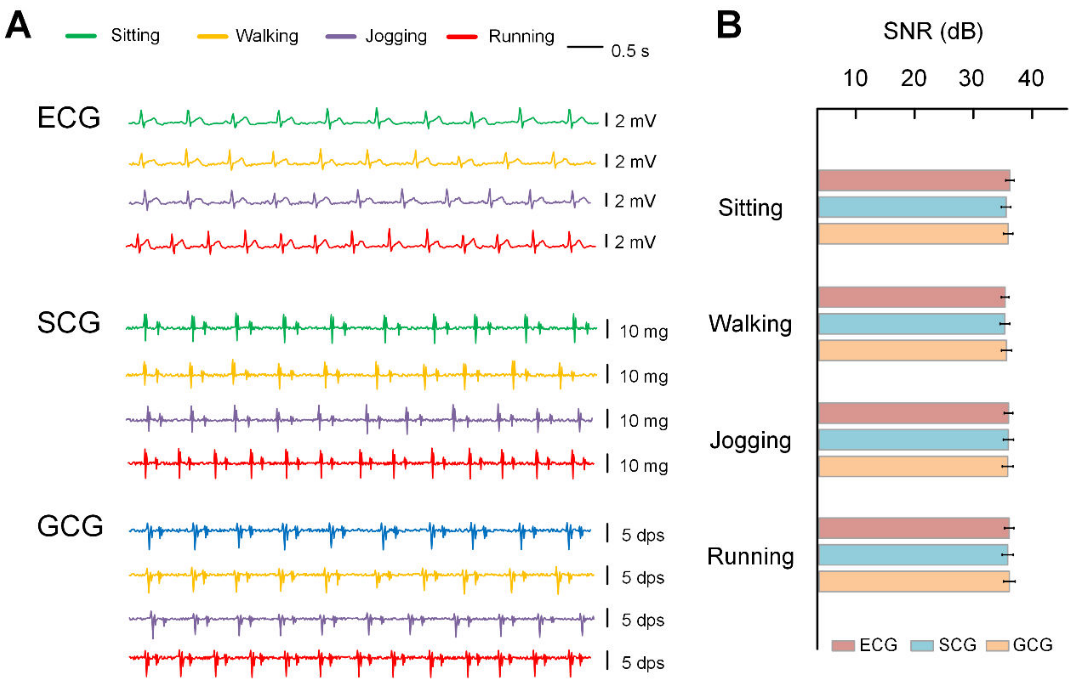

Fig. S20. Output performance of the starfish-like device. (**A**) ECG, SCG, and GCG signals recorded from a subject during sitting, walking, jogging, and running. (**B**) SNR of the collected ECG, SCG, and GCG signals using the starfish-like device, showing consistent stability across motion states, from sitting to running, with SNR values around 35 dB. These results demonstrate the effectiveness of the starfish-like wearable heart monitor in capturing high-fidelity cardiac electrical and mechanical signals during movement.

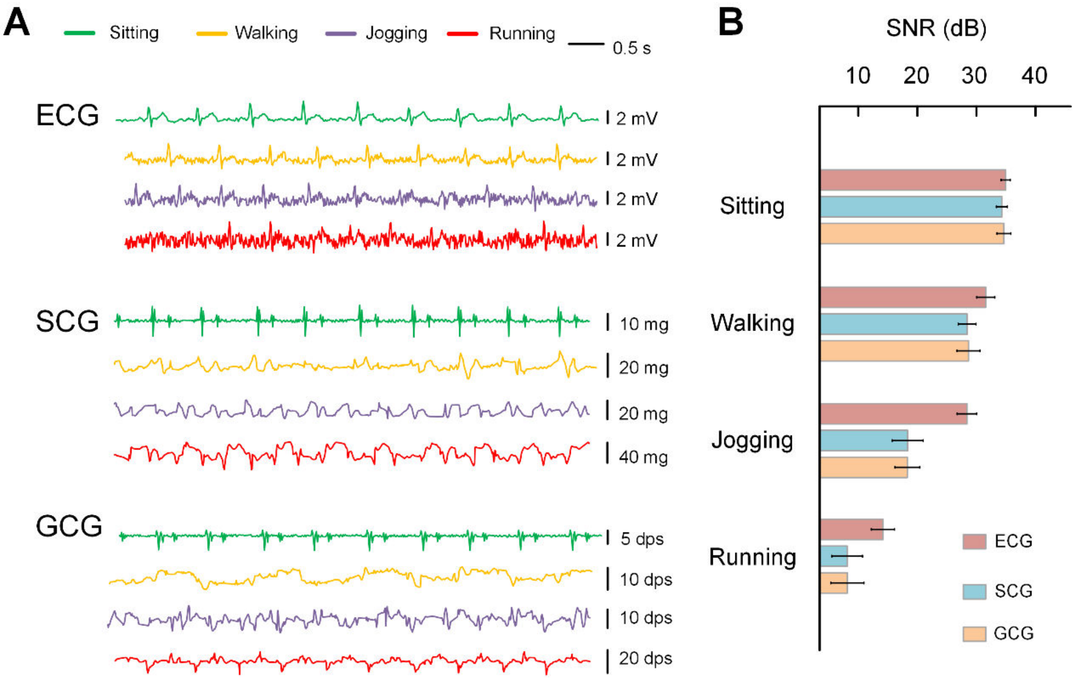

Fig. S21. Output performance of traditional monolithic wearable devices. (**A**) ECG, SCG, and GCG signals recorded from a subject during sitting, walking, jogging, and running. (**B**) SNR of the collected ECG, SCG, and GCG signals using traditional monolithic wearable devices, showing a decrease in SNR from around 35 dB while sitting to below 10 dB during running. This decline is attributed to the increasing impact of motion artifacts, which typically manifest as rhythmic patterns associated with movement.

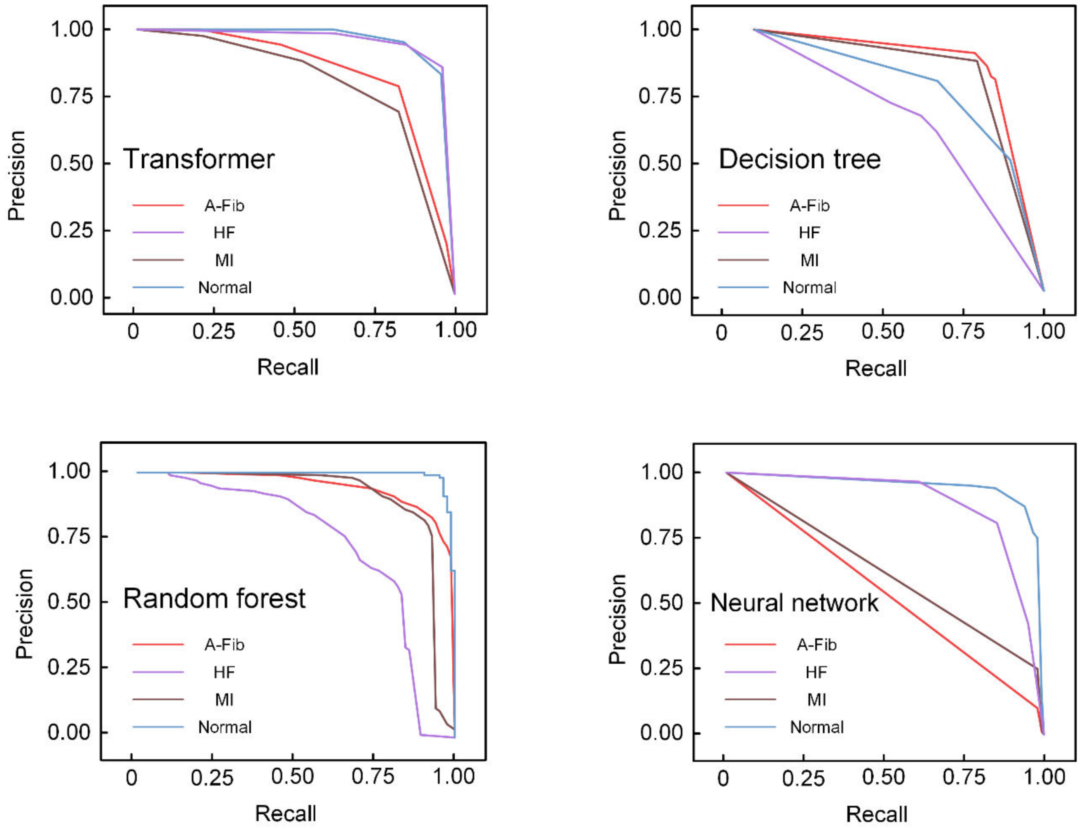

Fig. S22. Performance comparison of ML models for heart disease diagnosis. The transformer model outperforms other models, including decision tree, random forest, and neural network composed of fully-connected layers. Precision indicates the proportion of correctly identified positives, calculated as:

$$\text{Precision} = \frac{\text{TP}}{\text{TP} + \text{FP}}$$

where TP (True Positive) represents correct positive predictions, and FP (False Positive) represents incorrect positive predictions. A high precision score reflects a model's accuracy in predicting positive results.

Recall measures the proportion of actual positives that are correctly identified by the model, calculated as:

$$\text{Recall} = \frac{\text{TP}}{\text{TP} + \text{FN}}$$

where FN (False Negative) represents actual positives that were predicted as negatives by the model. A high recall indicates that the model is able to identify most of the actual positives. The Precision-Recall Curve shows the trade-off between precision and recall at various threshold levels. Each point on the curve represents a precision and recall value at a specific threshold. By adjusting the model's decision threshold, the interplay between precision and recall can be observed: typically, increasing precision leads to a decrease in recall, and vice versa.

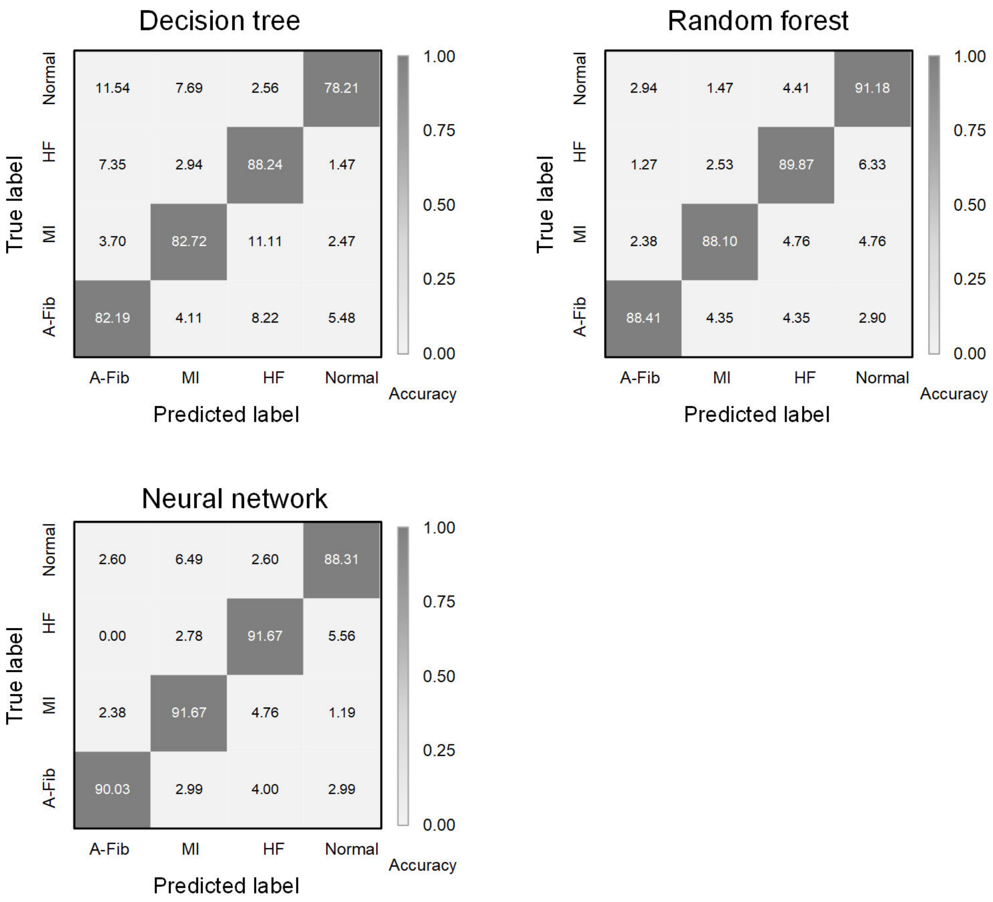

Fig. S23. Confusion matrix of different models displaying classification accuracy for predicting each type of cardiac disease in the test set. Using features extracted from cardiac mechanical and electrical signals, the Decision Tree model achieved accuracies of 78.21% for normal, 88.24% for HF, 82.72% for MI, and 82.19% for A-Fib. In comparison, the Random Forest model achieved accuracies of 91.18% for normal, 89.87% for HF, 88.10% for MI, and 88.41% for A-Fib. Meanwhile, the Neural Network model recorded accuracies of 88.31% for normal, 91.67% for HF, 91.67% for MI, and 90.03% for A-Fib.

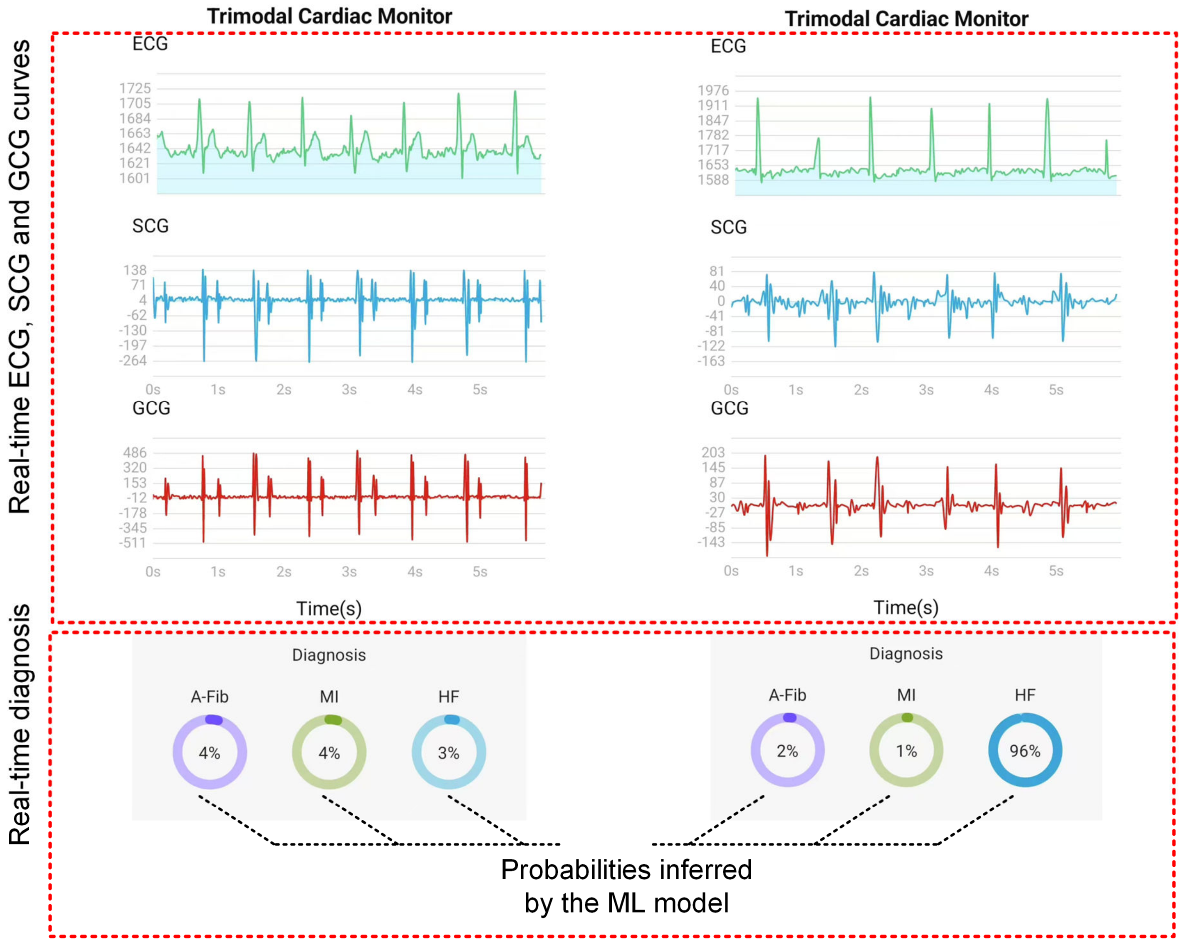

Fig. S24. Screenshots of the starfish-like device interfaces from a normal user (left) and a heart failure (HF) patient (right). During use, the starfish-like device displays real-time ECG, SCG, and GCG curves, along with heart disease classification probabilities, reflecting the wearer's heart health or disease status on mobile clients (see movie S3 and movie S4).

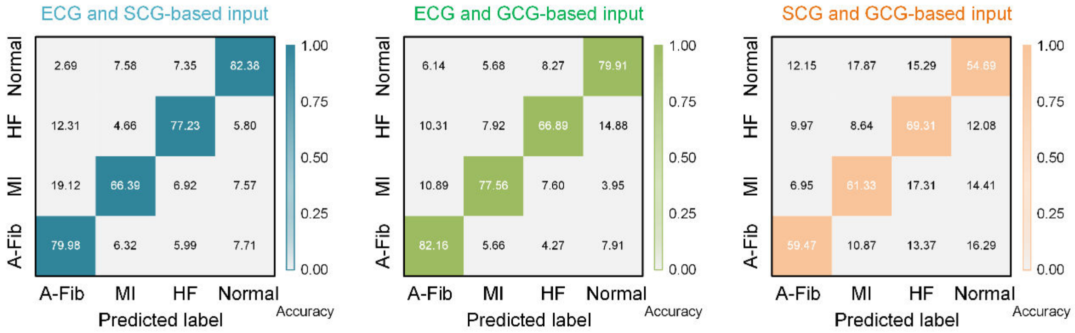

Fig. S25. Confusion matrix comparing classification performance using various combinations of ECG, SCG, or GCG signals. The results demonstrate a notable improvement in accuracy when using dual-signal combinations compared to single-signal inputs. However, the accuracy remains lower than that achieved with the combined use of all three signals (ECG, SCG, and GCG) as input.

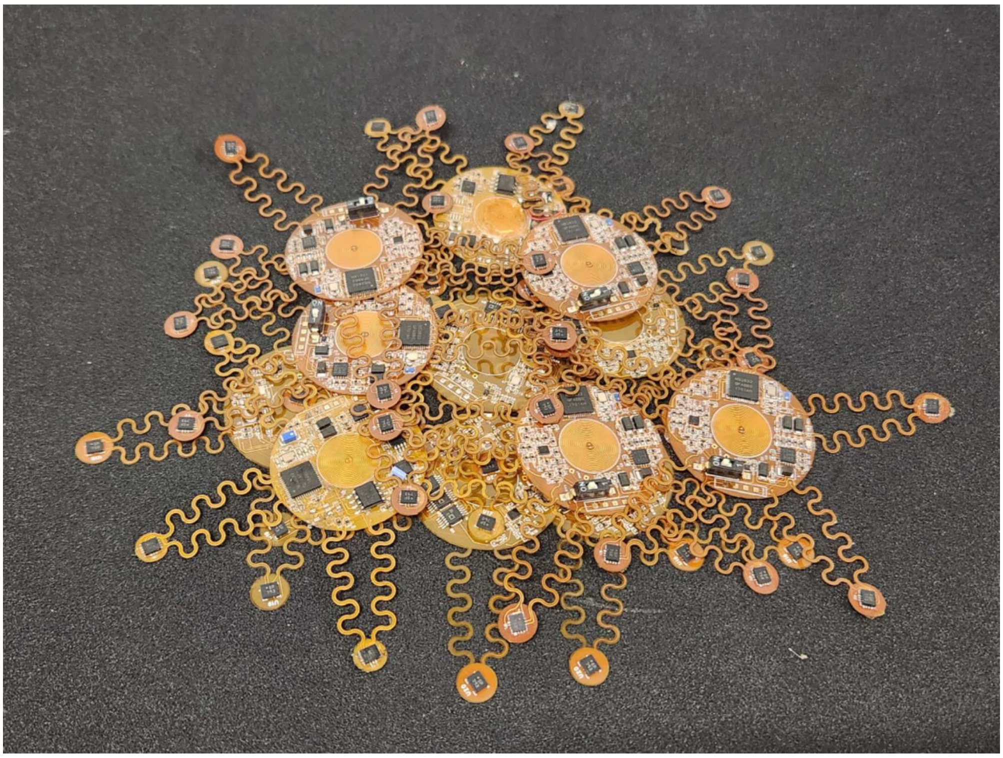

Fig. S26. Mass-produced starfish-like devices using the standardized flexible printed circuit board fabrication process.

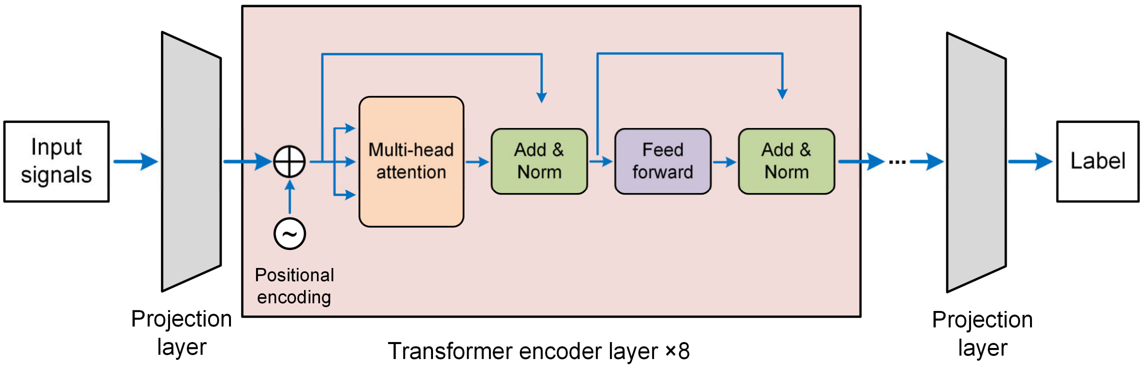

Fig. S27. The Transformer model architecture for both disease diagnosis and motion recognition tasks. The model processes input signals through a projection layer, followed by eight Transformer encoder layers, another projection layer, and finally a classification loss function.

Table S1. Comparison of the starfish-inspired device with state-of-the-art heart monitors.

<table>
<tr><th rowspan="2">Device configuration</th><th colspan="2">Cardiac parameters</th><th rowspan="2">Activities</th><th rowspan="2">Signal quality</th><th rowspan="2">Funtions</th><th colspan="2">Human studies</th></tr>
<tr><th>Electrical</th><th>Mechanical</th><th>Healthy</th><th>Patients</th></tr>
<tr><td>Starfishtronics (our work)
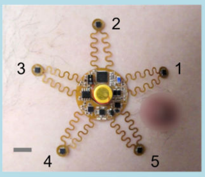</td><td>ECG</td><td>SCG & GCG</td><td rowspan="2">Sitting, walking, jogging and running</td><td rowspan="2">High-fidelity cardiac electrical and mechanical signals (SNR: ~35 dB) are captured across different motion states.</td><td rowspan="2">A highly integrated, lightweight (~1.7 g), and waterproof wearable system capable of real-time signal acquisition, in-situ data processing, edge computing using deployed ML models, wireless data transmission, wireless charging and powering.</td><td>16</td><td>18</td></tr>
<tr><td></td><td colspan="2">(Additional cardiac parameters, including HR, QT, SDRR, EMD, PEP, and LVET, can also be derived from ECG, SCG, and GCG waveforms.)</td><td colspan="2">Tested on both healthy individuals and patients with cardiac diseases, achieving 92% accuracy in diagnosing various heart conditions, including A-Fib, MI, and HF.</td></tr>
<tr><td>Monolithic device (10)
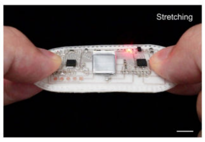</td><td>ECG</td><td>No</td><td rowspan="2">Stationary, during motion, and after motion</td><td rowspan="2">High-quality ECG signals are captured before and after motion, with compromised signals during motion.</td><td rowspan="2">A highly integrated, waterproof wearable system with real-time signal acquisition and wireless data transmission capabilities.</td><td>12</td><td>No</td></tr>
<tr><td></td><td colspan="2">(Other cardiac parameters, such as HR, QT, and SDRR, are obtained from ECG waveforms.)</td><td colspan="2">Healthy subjects used for device testing.</td></tr>
<tr><td>Monolithic device (23)
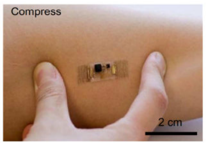</td><td>ECG</td><td>SCG</td><td rowspan="2">Stationary</td><td rowspan="2">Stable ECG and SCG signals are achieved under stationary conditions.</td><td rowspan="2">The wearable device includes only electrodes and inertial measurement unit (IMU) components, which are connected to external equipment.</td><td>Yes</td><td>8</td></tr>
<tr><td></td><td colspan="2">(Acoustic analysis is performed for further applications.)</td><td colspan="2">Healthy subjects were used for device testing, while patients were involved for demonstrating abnormal signals.</td></tr>
<tr><td>Monolithic device (11)
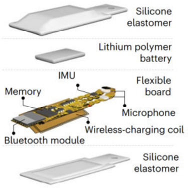</td><td>ECG</td><td>SCG</td><td rowspan="2">Stationary, and body movements</td><td rowspan="2">Stable ECG and SCG signals are obtained under stationary conditions.</td><td rowspan="2">A highly integrated wearable system with real-time signal acquisition, data processing, wireless data transmission, and remote charging capabilities.</td><td>Yes</td><td>Yes</td></tr>
<tr><td></td><td colspan="2">(Other cardiac parameters, such as HR, can be obtained.)</td><td colspan="2">Healthy subjects were used for device testing, while lung patients were involved to demonstrate abnormal lung sound signals using IMU components.</td></tr>
<tr><td>Monolithic device (27)
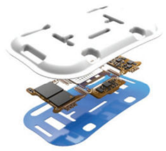</td><td>ECG</td><td>SCG</td><td rowspan="2">Stationary, laughing, talking, and walking</td><td rowspan="2">Stable ECG and SCG signals are obtained during sitting, with compromised signals during motion.</td><td rowspan="2">A highly integrated wearable system featuring real-time signal acquisition, data processing, wireless data transmission, and remote charging.</td><td>Yes</td><td>Yes</td></tr>
<tr><td></td><td colspan="2">(Other cardiac parameters, such as HR and RR, can be captured using the device.)</td><td colspan="2">Healthy subjects were used for device testing, with a neonatal patient for comparison.</td></tr>
<tr><td>Wire-connected device (66)
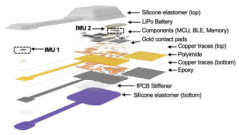</td><td>No</td><td>No</td><td rowspan="2">Stationary, eating, talking, drinking, etc.</td><td rowspan="2">Stable respiratory and swallowing signals are achieved under various conditions.</td><td rowspan="2">A highly integrated wearable system featuring real-time signal acquisition, data processing, wireless data transmission, and remote charging.</td><td>67</td><td>4</td></tr>
<tr><td></td><td colspan="2">(Other parameters, such as respiratory phase and swallowing events, can be obtained using this device.)</td><td colspan="2">Validation studies in healthy adults and patients with dysphagia.</td></tr>
</table>

Table S1. Comparison of the starfish-inspired device with state-of-the-art heart monitors.

Device configuration	Cardiac parameters		Activities	Signal quality	Funtions	Human subjects	
	Electrical	Mechanical				Healthy	Patients
Wire-connected device (30)<br>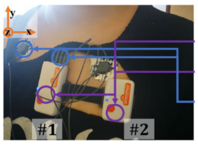 	ECG<br>(Other cardiac parameters, such as cardiac intervals, are obtained from ECG, SCG, and GCG waveforms.)	SCG & GCG	Stationary, walking, and jogging	Stable signals are obtained during stationary conditions. Only cardiac intervals are analyzed during motion.	Commercial ECG electrodes and IMU sensors are wired to external bulky equipment.	4<br>Healthy subjects were used for device testing.	No
Wire-connected device (67)<br>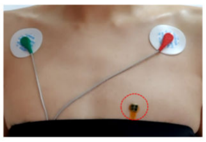	ECG<br>(Other cardiac parameters, such as HR, can be obtained from ECG and SCG waveforms.)	SCG	Stationary	Stable ECG and SCG signals can be achieved under stationary conditions.	Commercial ECG electrodes and IMU sensors are connected to external bulky equipment via wires.	16<br>Healthy subjects were used for device testing.	No
Monolithic device (20)<br>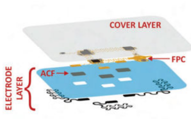 	ECG<br>(Other cardiac parameters, such as PEP and LVET, are obtained from ECG and SCG waveforms.)	SCG	Stationary, and after exercise	Stable ECG and SCG signals are achieved during and after motion.	A highly integrated wearable system with capabilities for signal acquisition, processing, and wireless transmission.	Yes<br>Healthy subjects were used for device testing.	No
Monolithic device (22)<br>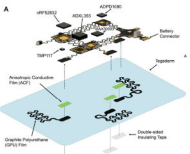	ECG<br>(Other cardiac parameters, such as PEP and LVET, are obtained from ECG and SCG waveforms.)	SCG	Stationary, walking, and jogging	Recovered signals are achieved during specific motions.	A highly integrated wearable system with functionalities for signal acquisition, processing, and wireless transmission.	Yes<br>Healthy subjects were used for device testing.	No
Monolithic device (24)<br>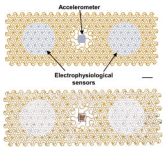 	ECG<br>(Other cardiac parameters, such as peak information, are obtained from ECG and SCG waveforms.)	SCG	Stationary	Stable ECG and SCG signals are obtained under stationary conditions.	Electrodes and IMU components on a porous substrate with functionalities for signal acquisition, processing, and wireless transmission.	Yes<br>Healthy subjects were used for device testing.	No

Notes: For many state-of-the-art wearable devices, signal-to-noise ratio (SNR) during motion is not provided, but their performance is generally known to degrade due to the motion artifacts (*68*).

Table S2. Off-the-shelf electronic components used in the starfish-like device.

Components	Model/Value	Description
C1, C14	100 nF	Power filter capacitor
C2	1 uF	Signal filter capacitor
C3, C6	1 uF	Power filter capacitor
C4, C5,	12 pF	Load Capacitors
C7,	100 pF	Coupling Capacitor
C8,	0.8 pF	Tuning Capacitors
C9	1.2 pF	Tuning Capacitors
C10	4.7 uF	Charge Pump Capacitor
C11	10 uF	Input Decoupling Capacitor
C12, C13,	22 uF	Decoupling Capacitors
C15	470 nF	Coupling Capacitor
R1	2.05 KΩ	Gain Setting Resistor
R2, R3	47 KΩ	Feedback/Biasing Resistors
R4	2 MΩ	Input Impedance Resistor
R5	1 MΩ	High-Value Resistor
R6, R7	4.7 KΩ	Pull-up Resistors
SW1	DSHP01TSGER	Power switch
L1	10 uH	Out Low-Frequency Filtering
L2	15 nH	High-Frequency Filtering
L3	3.9 nH	Tuning Inductor
X1	32 MHz	Frequency Generator
U1	NRF52832	Bluetooth and Controller
U2	TPS7A2033PDQNR	Voltage Regulator
U3	ANT016008LCS2442MA2	Signal Transmission
U4	LM7705MMX	Negative Voltage Generator
U5	INA333	Instrumentation Amplifier
U6	OPA2333	Operational Amplifier
U7 – U11	BMI270	Inertial Measurement Unit

Movie S1.

Mechanical vibration experiments with various device configurations. Experimental setup with the device placed on artificial skin and a vibration motor positioned at one end. The motor is controlled by voltage toggling, and displacement curves of each sensing element are collected during operation (fig. S1). The results show that the five-arm configuration significantly reduces mechanical coupling compared to the other designs.

Movie S2.

Wireless charging and powering for the starfish-like device. The remote power management system not only recharges the device battery in various conditions, such as using a commercial charger, during normal wear, and even underwater, but can also supply power directly to the device without a battery, ensuring its continued operation (fig. S5). It charges efficiently in close proximity to commercial wireless chargers or by harvesting energy from nearby wireless charging coil during operation, ensuring continuous, long-lasting functionality without the need for battery replacement.

Movie S3.

Trimodal cardiac monitoring with a monolithic device and the starfish-like device during various physical activities. Even during various motion sates, the recorded cardiac biosignals maintain high signal-to-noise ratios comparable to that of a stationary state, while clearly preserving distinct feature peaks (fig. S19). This ability to decouple motion artifacts is not only a result of signal compensation and motion-adaptive filtering but also due to the bioinspired starfish-like device configuration.

Movie S4.

Cardiac disease diagnosis using ultrasound imaging during static conditions and the starfish-like device during motions. Current clinical ultrasound devices enable the diagnosis of cardiac diseases by assessing the mechanical activity of the heart to determine its pumping ability. However, these assessments typically require patients to visit hospitals and remain in a stationary state during examinations. In contrast, the starfish-like device allows for continuous monitoring without disrupting the patient's daily activities. With its embedded machine learning model for disease diagnosis, it can provide high-accuracy alerts for cardiac conditions while the user is wearing the device.

REFERENCES AND NOTES

1. L. M. Blowes, M. Egertová, Y. Liu, G. R. Davis, N. J. Terrill, H. S. Gupta, M. R. Elphick, Body wall structure in the starfish Asterias rubens. *J. Anat.* **231**, 325–341 (2017).

2. P. O'Neill, Structure and mechanics of starfish body wall. *J. Exp. Biol.* **147**, 53–89 (1989).

3. D.-H. Kim, N. Lu, R. Ma, Y.-D. Kim, R.-H. Kim, S. Wang, J. Wu, S. M. Won, H. Tao, A. Islam, K. J. Yu, T.-i. Kim, R. Chowdhury, M. Ying, L. Xu, M. Li, H.-J. Chung, H. Keum, M. McCormick, P. Liu, Y.-W. Zhang, F. G. Omenetto, Y. Huang, T. Coleman, J. A. Rongers, Epidermal electronic. *Science* **333**, 838–843 (2011).

4. Z. Liu, X. Hu, R. Bo, Y. Yang, X. Cheng, W. Pang, Q. Liu, Y. Wang, S. Wang, S. Xu, Z. Shen, Y. Zhang, A three-dimensionally architected electronic skin mimicking human mechanosensation. *Science* **384**, 987–994 (2024).

5. Y. Huang, J. Zhou, P. Ke, X. Guo, C. K. Yiu, K. Yao, S. Cai, D. Li, Y. Zhou, J. Li, T. H. Wong, Y. Liu, L. Li, Y. Gao, X. Huang, H. Li, J. Li, B. Zhang, Z. Chen, H. Zheng, X. Yang, H. Gao, Z. Zhao, X. Guo, E. Song, H. Wu, Z. Wang, Z. Xie, K. Zhu, X. Yu, A skin-integrated multimodal haptic interface for immersive tactile feedback. *Nat. Electron.* **6**, 1020–1031 (2023).

6. Z. Yan, D. Xu, Z. Lin, P. Wang, B. Cao, H. Ren, F. Song, C. Wan, L. Wang, J. Zhou, X. Zhao, J. Chen, Y. Huang, X. Duan, Highly stretchable van der Waals thin films for adaptable and breathable electronic membranes. *Science* **375**, 852–859 (2022).

7. A. Libanori, G. Chen, X. Zhao, Y. Zhou, J. Chen, Smart textiles for personalized healthcare. *Nat. Electron.* **5**, 142–156 (2022).

8. H. Shim, K. Sim, B. Wang, Y. Zhang, S. Patel, S. Jang, T. J. Marks, A. Facchetti, C. Yu, Elastic integrated electronics based on a stretchable n-type elastomer–semiconductor-elastomer stack. *Nat. Electron.* **6**, 349–359 (2023).

9. H. U. Chung, B. H. Kim, J. Y. Lee, J. Lee, Z. Xie, E. M. Ibler, K. Lee, A. Banks, J. Y. Jeong, J. Kim, C. Ogle, D. Grande, Y. Yu, H. Jang, P. Assem, D. Ryu, J. W. Kwak, M. Namkoong, J. B.

Park, Y. Lee, D. H. Kim, A. Ryu, J. Jeong, K. You, B. Ji, Z. Liu, Q. Huo, X. Feng, Y. Deng, Y. Xu, K.-I. Jang, J. Kim, Y. Zhang, R. Ghaffari, C. M. Rand, M. Schau, A. Hamvas, D. E. Weese-Mayer, Y. Huang, S. M. Lee, C. H. Lee, N. R. Shanbhag, A. S. Paller, S. Xu, J. A. Rogers, Binodal, wireless epidermal electronic systems with in-sensor analytics for neonatal intensive care. *Science* **363**, eaau0780 (2019).

10. B. Zhang, J. Li, J. Zhou, L. Chow, G. Zhao, Y. Huang, Z. Ma, Q. Zhang, Y. Yang, C. Yiu, J. Li, F. Chun, X. Huang, Y. Gao, P. Wu, S. Jia, H. Li, D. Li, Y. Liu, K. Yao, R. Shi, Z. Chen, B. L. Khoo, W. Yang, F. Wang, Z. Zheng, Z. Wang, X. Yu, A three-dimensional liquid diode for soft, integrated permeable electronics. *Nature* **628**, 84–92 (2024).

11. J.-Y. Yoo, S. Oh, W. Shalish, W.-Y. Maeng, E. Cerier, E. Jeanne, M.-K. Chung, S. Lv, Y. Wu, S. Yoo, A. Tzavelis, J. Trueb, M. Park, H. Jeong, E. Okunzuwa, S. Smilkova, G. Kim, J. Kim, G. Chung, Y. Park, A. Banks, S. Xu, G. M. Sant'Anna, D. E. Weese-Mayer, A. Bharat, J. A. Rogers, Wireless broadband acousto-mechanical sensing system for continuous physiological monitoring. *Nat. Med.* **29**, 3137–3148 (2023).

12. T. R. Ray, J. Choi, A. J. Bandodkar, S. Krishnan, P. Gutruf, L. Tian, R. Ghaffari, J. A. Rogers, Bio-integrated wearable systems: A comprehensive review. *Chem. Rev.* **119**, 5461–5533 (2019).

13. J. Kim, A. S. Campbell, B. E.-F. de Ávila, J. Wang, Wearable biosensors for healthcare monitoring. *Nat. Biotechnol.* **37**, 389–406 (2019).

14. Y. J. Hong, H. Jeong, K. W. Cho, N. Lu, D. H. Kim, Wearable and implantable devices for cardiovascular healthcare: From monitoring to therapy based on flexible and stretchable electronics. *Adv. Funct. Mater.* **29**, 1808247 (2019).

15. N. Khaltaev, S. Axelrod, Countrywide cardiovascular disease prevention and control in 49 countries with different socio-economic status. *Chronic Dis. Transl. Med.* **8**, 296–304 (2022).

16. P. Ponikowski, S. D. Anker, K. F. AlHabib, M. R. Cowie, T. L. Force, S. Hu, T. Jaarsma, H. Krum, V. Rastogi, L. E. Rohde, U. C. Samal, H. Shimokawa, B. B. Siswanto, K. Sliwa, G. Filippatos, Heart failure: Preventing disease and death worldwide. *ESC Heart Fail.* **1**, 4–25 (2014).

17. Y. S. Choi, H. Jeong, R. T. Yin, R. Avila, A. Pfenniger, J. Yoo, J. Y. Lee, A. Tzavelis, Y. J. Lee, S. W. Chen, H. S. Knight, S. Kim, H.-Y. Ahn, G. Wickerson, A. Vázquez-Guardado, E. Higbee-Dempsey, B. A. Russo, M. A. Napolitano, T. J. Holleran, L. A. Razzak, A. N. Miniovich, G. Lee, B. Geist, B. Kim, S. Han, J. A. Brennan, K. Aras, S. S. Kwak, J. Kim, E. A. Waters, X. Yang, A. Burrell, K. S. Chun, C. Liu, C. Wu, A. Y. Rwei, A. N. Spann, A. Banks, D. Johnson, Z. J. Zhang, C. R. Haney, S. H. Jin, A. V. Sahakian, Y. Huang, G. D. Trachiotis, B. P. Knight, R. K. Arora, I. R. Efimov, J. A. Rogers, A transient, closed-loop network of wireless, body-integrated devices for autonomous electrotherapy. *Science* **376**, 1006–1012 (2022).

18. S. K. Gill, A. Barsky, X. Guan, K. V. Bunting, A. Karwath, O. Tica, M. Stanbury, S. Haynes, A. Folarin, R. Dobson, J. Kurps, F. W. Asselbergs, D. E. Grobbee, A. J. Camm, M. J. C. Eijkemans, G. V. Gkoutos, D. Kotecha, BigData@Heart Consortium, cardAIc group, RATE-AF trial team, Consumer wearable devices for evaluation of heart rate control using digoxin versus beta-blockers: The RATE-AF randomized trial. *Nat. Med.* **30**, 2030–2036 (2024).

19. J. H. Koo, S. Jeong, H. J. Shim, D. Son, J. Kim, D. C. Kim, S. Choi, J.-I. Hong, D.-H. Kim, Wearable electrocardiogram monitor using carbon nanotube electronics and color-tunable organic light-emitting diodes. *ACS Nano* **11**, 10032–10041 (2017).

20. S. Bhattacharya, M. Nikbakht, A. Alden, P. Tan, J. Wang, T. A. Alhalimi, S. Kim, P. Wang, H. Tanaka, A. Tandon, E. F. Coyle, O. T. Inan, N. Lu, A Chest-conformable, wireless electro-mechanical E-tattoo for measuring multiple cardiac time intervals. *Adv. Electron. Mater.* **9**, 2201284 (2023).

21. H. U. Chung, A. Y. Rwei, A. Hourlier-Fargette, S. Xu, K. Lee, E. C. Dunne, Z. Xie, C. Liu, A. Carlini, D. H. Kim, D. Ryu, E. Kulikova, J. Cao, I. C. Odland, K. B. Fields, B. Hopkins, A. Banks, C. Ogle, D. Grande, J. B. Park, J. Kim, M. Irie, H. Jang, J. Lee, Y. Park, J. Kim, H. H. Jo, H. Hahm, R. Avila, Y. Xu, M. Namkoong, J. W. Kwak, E. Suen, M. A. Paulus, R. J. Kim, B. V. Parsons, K. A. Human, S. S. Kim, M. Patel, W. Reuther, H. S. Kim, S. H. Lee, J. D. Leedle, Y. Yun, S. Rigali, T. Son, I. Jung, H. Arafa, V. R. Soundararajan, A. Ollech, A. Shukla, A. Bradley, M. Schau, C. M. Rand, L. E. Marsillio, Z. L. Harris, Y. Huang, A. Hamvas, A. S. Paller, D. E. Weese-Mayer, J. Y. Lee, J. A. Rogers, Skin-interfaced biosensors

for advanced wireless physiological monitoring in neonatal and pediatric intensive-care units. *Nat. Med.* **26**, 418–429 (2020).

22. S. Bhattacharya, F. Santucci, M. Jankovic, T. Huang, J. Basu, P. Tan, E. Schena, N. Lu, Cardiac Time Intervals under Motion Using Bimodal Chest E-Tattoos and Multistage Processing. *IEEE Trans. Biomed. Eng.* **72**, 413–424 (2024).

23. Y. Liu, J. J. S. Norton, R. Qazi, Z. Zou, K. R. Ammann, H. Liu, L. Yan, P. L. Tran, K.-I. Jang, J. W. Lee, D. Zhang, K. A. Kilian, S. H. Jung, T. Bretl, J. Xiao, M. J. Slepian, Y. Huang, J.-W. Jeong, J. A. Rogers, Epidermal mechano-acoustic sensing electronics for cardiovascular diagnostics and human-machine interfaces. *Sci. Adv.* **2**, e1601185 (2016).

24. Y. Ling, G. Zhao, Y. Su, Q. Wu, Y. Xu, Z. Chen, B. Arends, O. Emeje, G. Huang, J. Xie, Z. Yan, Skin-inspired porous mesh bioelectronics with built-in multifunctionality for concurrently monitoring heart electrical and mechanical functions. *Adv. Funct. Mater.* **33**, 2302681 (2023).

25. T. Ha, J. Tran, S. Liu, H. Jang, H. Jeong, R. Mitbander, H. Huh, Y. Qiu, J. Duong, R. L. Wang, P. Wang, A. Tandon, J. Sirohi, N. Lu, A chest-laminated ultrathin and stretchable E-tattoo for the measurement of electrocardiogram, seismocardiogram, and cardiac time intervals. *Adv. Sci.* **6**, 1900290 (2019).

26. F. Ershad, A. Thukral, J. Yue, P. Comeaux, Y. Lu, H. Shim, K. Sim, N.-I. Kim, Z. Rao, R. Guevara, L. Contreras, F. Pan, Y. Zhang, Y.-S. Guan, P. Yang, X. Wang, P. Wang, X. Wu, C. Yu, Ultra-conformal drawn-on-skin electronics for multifunctional motion artifact-free sensing and point-of-care treatment. *Nat. Commun.* **11**, 3823 (2020).

27. S. S. Kwak, S. Yoo, R. Avila, H. U. Chung, H. Jeong, C. Liu, J. L. Vogl, J. Kim, H.-J. Yoon, Y. Park, H. Ryu, G. Lee, J. Kim, J. Koo, Y. S. Oh, S. Kim, S. Xu, Z. Zhao, Z. Xie, Y. Huang, J. A. Rogers, Skin-integrated devices with soft, holey architectures for wireless physiological monitoring, with applications in the neonatal intensive care unit. *Adv. Mater.* **33**, 2103974 (2021).

28. C. Liu, J.-T. Kim, D. S. Yang, D. Cho, S. Yoo, S. R. Madhvapathy, H. Jeong, T. Yang, H. Luan, R. Avila, J. Park, Y. Wu, K. Bryant, M. Cho, J. Lee, J. Y. Kwak, W. Ryu, Y. Huang, R.

G. Nuzzo, J. A. Rogers, Multifunctional materials strategies for enhanced safety of wireless, skin-interfaced bioelectronic devices. *Adv. Funct. Mater.* **33**, 2302256 (2023).

29. S.-H. Sunwoo, S. I. Han, C. S. Park, J. H. Kim, J. S. Georgiou, S.-P. Lee, D.-H. Kim, T. Hyeon, Soft bioelectronics for the management of cardiovascular diseases. *Nat. Rev. Bioeng.* **2**, 8–24 (2024).

30. C. Yang, N. Tavassolian, An independent component analysis approach to motion noise cancelation of cardio-mechanical signals. *IEEE Trans. Biomed. Eng.* **66**, 784–793 (2018).

31. S. Choi, S. I. Han, D. Jung, H. J. Hwang, C. Lim, S. Bae, O. K. Park, C. M. Tschabrunn, M. Lee, S. Y. Bae, J. W. Yu, J. H. Ryu, S.-W. Lee, K. Park, P. M. Kang, W. B. Lee, R. Nezafat, T. Hyeon, D.-H. Kim, Highly conductive, stretchable and biocompatible Ag-Au core-sheath nanowire composite for wearable and implantable bioelectronics. *Nat. Nanotechnol.* **13**, 1048–1056 (2018).

32. R. J. Oweis, B. O. Al-Tabbaa, QRS detection and heart rate variability analysis: A survey. *Biomed. Sci. Eng.* **2**, 13–34 (2014).

33. P. Reant, M. Dijos, E. Donal, A. Mignot, P. Ritter, P. Bordachar, P. Dos Santos, C. Leclercq, R. Roudaut, G. Habib, S. Lafitte, Systolic time intervals as simple echocardiographic parameters of left ventricular systolic performance: Correlation with ejection fraction and longitudinal two-dimensional strain. *Eur. J. Echocardiogr.* **11**, 834–844 (2010).

34. M. Klum, M. Urban, T. Tigges, A.-G. Pielmus, A. Feldheiser, T. Schmitt, R. Orglmeister, Wearable cardiorespiratory monitoring employing a multimodal digital patch stethoscope: Estimation of ECG, PEP, LVET and respiration using a 55 mm single-lead ECG and phonocardiogram. *Sensors* **20**, 2033 (2020).

35. Y. Nakamura, T. Konishi, H. Nonogi, T. Sakurai, S. Sasayama, C. Kawai, Myocardial relaxation in atrial fibrillation. *J. Am. Coll. Cardiol.* **7**, 68–73 (1986).

36. P. M. L. Janssen, Myocardial contraction-relaxation coupling. *Am. J. Physiol. Heart Circ. Physiol.* **299**, H1741–H1749 (2010).

37. H. Fukuta, W. C. Little, The cardiac cycle and the physiologic basis of left ventricular contraction, ejection, relaxation, and filling. *Heart Fail. Clin.* **4**, 1–11 (2008).

38. M. Cikes, L. Tong, G. R. Sutherland, J. D'hooge, Ultrafast cardiac ultrasound imaging: Technical principles, applications, and clinical benefits. *JACC Cardiovasc. Imaging* **7**, 812–823 (2014).

39. C. H. Davies, K. Davia, J. G. Bennett, J. R. Pepper, P. A. Poole-Wilson, S. E. Harding, Reduced contraction and altered frequency response of isolated ventricular myocytes from patients with heart failure. *Circulation* **92**, 2540–2549 (1995).

40. A. Alwaidh, M. Sharp, P. French, Laser processing of rigid and flexible PCBs. *Opt. Lasers Eng.* **58**, 109–113 (2014).

41. J. Govaerts, W. Christiaens, E. Bosman, J. Vanfleteren, Fabrication processes for embedding thin chips in flat flexible substrates. *IEEE Trans. Adv. Packag* **32**, 77–83 (2009).

42. O. Dietrich, J. G. Raya, S. B. Reeder, M. F. Reiser, S. O. Schoenberg, Measurement of signal-to-noise ratios in MR images: Influence of multichannel coils, parallel imaging, and reconstruction filters. *J. Magn. Reson. Imaging* **26**, 375–385 (2007).

43. Y. Sun, K. L. Chan, S. M. Krishnan, Characteristic wave detection in ECG signal using morphological transform. *BMC Cardiovasc. Disord.* **5**, 1–7 (2005).

44. E. Pueyo, J. P. Martínez, P. Laguna, Cardiac repolarization analysis using the surface electrocardiogram. *Philos. Trans. R. Soc. A.* **367**, 213–233 (2009).

45. S. Sieciński, P. S. Kostka, E. J. Tkacz, Gyrocardiography: A review of the definition, history, waveform description, and applications. *Sensors* **20**, 6675 (2020).

46. D. Rai, H. K. Thakkar, S. S. Rajput, J. Santamaria, C. Bhatt, F. Roca, A comprehensive review on seismocardiogram: Current advancements on acquisition, annotation, and applications. *Mathematics* **9**, 2243 (2021).

47. P. K. Sahoo, H. K. Thakkar, W.-Y. Lin, P.-C. Chang, M.-Y. Lee, On the design of an efficient cardiac health monitoring system through combined analysis of ECG and SCG signals. *Sensors* **18**, 379 (2018).

48. P. Dehkordi, K. Tavakolian, M. J. Tadi, V. Zakeri, F. Khosrow-Khavar, Investigating the estimation of cardiac time intervals using gyrocardiography. *Physiol. Meas.* **41**, 055004 (2020).

49. A. L. Goldberger, Z. D. Goldberger, A. Shvilkin, *Goldberger's Clinical Electrocardiography A Simplified Approach* (Elsevier, 2018).

50. R. Prabhu, I. D'Cruz, H. C. Cohen, G. Glick, Echocardiographic correlates of atrial contraction in normal and abnormal atrial rhythm. *Prog. Cardiovasc. Dis.* **20**, 463–478 (1978).

51. J. S. Meisner, D. M. McQueen, Y. Ishida, H. O. Vetter, U. Bortolotti, J. A. Strom, R. W. Frater, C. S. Peskin, E. L. Yellin, Effects of timing of atrial systole on LV filling and mitral valve closure: Computer and dog studies. *Am. J. Physiol. Heart Circ. Physiol.* **249**, H604–H619 (1985).

52. L. L. Konecke, H. Feigenbaum, S. Chang, B. C. Coray, J. C. Fischer, Abnormal mitral valve motion in patients with elevated left ventricular diastolic pressures. *Circulation* **47**, 989–996 (1973).

53. A. Shokouhmand, N.D. Aranoff, E. Driggin, P. Green, N. Tavassolian, Efficient detection ofaortic stenosis using morphological characteristics of cardiomechanical signals and heartrate variability parameters. Sci. Rep. 11, 23817 (2021).

54. M. Jafari Tadi, E. Lehtonen, A. Saraste, J. Tuminen, J. Koskinen, M. Teräs, J. Airaksinen, M. Pänkäälä, T. Koivisto, Gyrocardiography: A new non-invasive monitoring method for the assessment of cardiac mechanics and the estimation of hemodynamic variables. *Sci. Rep.* **7**, 6823 (2017).

55. S. Sieciński, P. S. Kostka, E. J. Tkacz, Heart rate variability analysis on electrocardiograms, seismocardiograms and gyrocardiograms on healthy volunteers. *Sensors* **20**, 4522 (2020).

56. S. Siecinski, P. S. Kostka, E. J. Tkacz, Time domain and frequency domain heart rate variability analysis on gyrocardiograms. *Annu. Int. Conf. IEEE Eng. Med. Biol. Soc.* **2020**, 2630–2633 (2020).

57. L. Sörnmo, P. Laguna, *Bioelectrical Signal Processing in Cardiac and Neurological Applications* (Academic Press, 2005).

58. P. R. Cavanagh, P. V. Komi, Electromechanical delay in human skeletal muscle under concentric and eccentric contractions. *Eur. J. Appl. Physiol. Occup. Physiol.* **42**, 159–163 (1979).

59. V. Gurev, J. Constantino, J. J. Rice, N. A. Trayanova, Distribution of electromechanical delay in the heart: Insights from a three-dimensional electromechanical model. *Biophys. J.* **99**, 745–754 (2010).

60. K. Russell, O. A. Smiseth, O. Gjesdal, E. Qvigstad, P. A. Norseng, I. Sjaastad, A. Opdahl, H. Skulstad, T. Edvardsen, E. W. Remme, Mechanism of prolonged electromechanical delay in late activated myocardium during left bundle branch block. *Am. J. Physiol. Heart Circ. Physiol.* **301**, H2334–H2343 (2011).

61. R. P. Paiva, P. Carvalho, R. Couceiro, J. Henriques, M. Antunes, I. Quintal, J. Muehlsteff, Beat-to-beat systolic time-interval measurement from heart sounds and ECG. *Physiol. Meas.* **33**, 177–194 (2012).

62. R. W. Clark, H. Boudoulas, S. F. Schaal, H. S. Schmidt, Adrenergic hyperactivity and cardiac abnormality in primary disorders of sleep. *Neurology* **30**, 113–119 (1980).

63. A. S. Alhakak, J. R. Teerlink, J. Lindenfeld, M. Böhm, G. M. C. Rosano, T. Biering-Sørensen, The significance of left ventricular ejection time in heart failure with reduced ejection fraction. *Eur. J. Heart Fail.* **23**, 541–551 (2021).

64. T. Biering-Sørensen, G. Querejeta Roca, S. M. Hegde, A. M. Shah, B. Claggett, T. H. Mosley Jr., K. R. Butler Jr., S. D. Solomon, Left ventricular ejection time is an independent predictor of incident heart failure in a community-based cohort. *Eur. J. Heart Fail.* **20**, 1106–1114 (2018).

65. A.-N. Marigliano, J.-T. Ortiz, J. Casas, A. Evangelista, Aortic regurgitation: From valvular to myocardial dysfunction. *J. Clin. Med.* **13**, 2929 (2024).

66. Y. J. Kang, H. M. Arafa, J.-Y. Yoo, C. Kantarcigil, J.-T. Kim, H. Jeong, S. Yoo, S. Oh, J. Kim, C. Wu, A. Tzavelis, Y. Wu, K. Kwon, J. Winograd, S. Xu, B. Martin-Harris, J. A. Rogers, Soft skin-interfaced mechano-acoustic sensors for real-time monitoring and patient feedback on respiratory and swallowing biomechanics. *NPJ Digit. Med.* **5**, 147 (2022).

67. S. Yu, S. Liu, A novel adaptive recursive least squares filter to remove the motion artifact in seismocardiography. *Sensors* **20**, 1596 (2020).

68. J. Yin, S. Wang, T. Tat, J. Chen, Motion artefact management for soft bioelectronics. *Nat. Rev. Bioeng.* **2**, 541–558 (2024).
